# Fully Humanized Bispecific T Cell Engager Shows Potent Activity in Central Nervous System and Peripheral Tumors

**DOI:** 10.1002/advs.202522391

**Published:** 2026-04-30

**Authors:** Joseph T. Duffy, Angela Martin‐Regalado, Benedikt E. Haupt, Jacob R. Pogue, Aditi Thakur, Manuel Fierro Cota, Markella Zannikou, Sol Misener, Vera P. Krymskaya, Kathleen McCortney, Jason Miska, Craig Horbinski, Dmitri Simberg, Maciej S. Lesniak, Charles D. James, Roger Stupp, Irina V. Balyasnikova

**Affiliations:** ^1^ Department of Neurological Surgery Northwestern University Chicago IL USA; ^2^ Northwestern Medicine Malnati Brain Tumor Institute of the Lurie Comprehensive Cancer Center Feinberg School of Medicine Northwestern University Chicago IL USA; ^3^ Department of Pathology Northwestern University Chicago IL USA; ^4^ Feinberg Cardiovascular and Renal Research Institute Northwestern University Chicago IL USA; ^5^ Department of Medicine Perelman School of Medicine University of Pennsylvania Philadelphia PA USA; ^6^ Department of Laboratory Medicine & Pathology Mayo Clinic Florida Jacksonville FL USA; ^7^ Department of Pharmaceutical Sciences Skaggs School of Pharmacy and Pharmaceutical Sciences Aurora CO USA; ^8^ Colorado Center for Nanomedicine and Nanosafety University of Colorado Anschutz Medical Campus Aurora CO USA

**Keywords:** brain, BTE, glioblastoma, IL13RA2, immunotherapy, metastases, solid tumors

## Abstract

Bispecific T cell engagers (BTEs) induce MHC‐independent cytotoxicity by bridging T cells to tumor cells via binding a T cell–activating receptor and a tumor‐associated antigen. BTEs have proven effective in hematologic malignancies and some solid tumors, yet their potential in glioblastoma (GBM) is largely unexplored. We developed a fully humanized BTE (hBTE) targeting interleukin‐13 receptor alpha 2 (IL13RA2), a tumor‐associated antigen widely expressed in GBM and associated with poor prognosis. In vitro, hBTE activated T cells and induced antigen‐dependent cytokine release and cytotoxicity against IL13RA2‐positive GBM cells. In vivo, hBTE showed robust target‐specific activity and markedly prolonged survival in primary and recurrent GBM xenograft models, without detectable off‐target local or systemic toxicity. Beyond GBM, hBTE also exhibited antitumor activity in IL13RA2‐expressing solid tumors, demonstrating selective tumor accumulation and therapeutic efficacy in models of breast cancer brain metastases and extracranial lung cancer. This work highlights the therapeutic potential of BTEs in IL13RA2‐expressing tumors and establishes a strong preclinical rationale for advancing hBTE therapy toward clinical translation in GBM and other tumors.

## Graphical abstract

This study reports the development of a fully humanized bispecific T cell engager targeting IL13RA2, a tumor‐associated antigen enriched in glioblastoma. This off‐the‐shelf immunotherapy drives potent, antigen‐dependent T cell activation and tumor killing, and prolongs survival in experimental GBM and other solid tumors models without detectable off‐target toxicity.

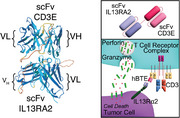

## 1 Introduction

Bispecific T cell engagers (BTEs) are off‐the‐shelf therapeutic engineered antibodies that redirect T cells to cancer. The clinical efficacy of BTEs has been demonstrated primarily in hematologic malignancies, with regulatory approval of agents such as blinatumomab [1, 2] and teclistamab [3, 4], while the application in solid tumors remains limited. Tebentafusp—a gp100‐targeting BTE—marked the first clinical success of this platform by improving survival in metastatic uveal melanoma [5]. In glioblastoma (GBM), the most frequent and aggressive primary brain tumor in adults, the median overall survival is around 15–17 months and a near‐universal recurrence rate despite surgery, radiotherapy (RT), temozolomide (TMZ), and tumor‐treating fields [6, 7, 8]. The poor outcome of this disease is driven in part by a profoundly immunosuppressive tumor microenvironment, characterized by myeloid cell dominance and limited, dysfunctional effector T cell responses. Together with antigen heterogeneity and immune evasion, these features underscore the need for strategies that engage and activate T cells within the tumor microenvironment, driving interest in T cell–redirecting therapies in GBM, particularly chimeric antigen receptor (CAR) T cell therapy and BTEs.

CAR T cell therapy, in which T cells are engineered to express tumor‐targeting receptors, has demonstrated the feasibility of targeting GBM‐associated antigens. However, autologous manufacturing complexity, prolonged vein‐to‐vein time, and the risk of neurotoxicity within the central nervous system have constrained its clinical application in GBM [9]. In contrast, BTEs are off‐the‐shelf therapeutics that do not require autologous, patient‐specific engineering [10]. Several preclinical studies have demonstrated the efficacy of EGFRvIII‐targeted BTEs [11, 12, 13], with a first‐in‐human clinical trial (NCT04903795) expected to begin in April 2026. BTEs comprise two single‐chain variable fragments (scFvs) fused into a single molecule: one targets a T cell‐activating receptor, such as CD3 epsilon (CD3E) or CD28, and the other binds a tumor antigen. By physically linking T cells to tumor cells, BTEs promote cytotoxic immune synapse formation and induce perforin‐ and granzyme‐mediated tumor cell killing. Unlike conventional T cell responses, which rely on MHC‐restricted presentation, BTE‐mediated activation occurs independently of MHC [14]. This feature is particularly advantageous in solid tumors, including gliomas, where loss of MHC expression is a well‐established mechanism of immune evasion [15, 16].

Our group developed a humanized BTE (hBTE) that targets human interleukin‐13 receptor alpha 2 (IL13RA2) and CD3E on the T cell receptor. Humanization mitigates immunogenicity and supports repeated systemic administration, which is critical for achieving sustained therapeutic activity and facilitating clinical translation [17, 18]. IL13RA2 is a tumor‐associated antigen expressed in 50%–80% of GBMs and largely absent from normal tissues, except the testes [19, 20, 21, 22]. Its expression is associated with higher tumor grade, the mesenchymal transcriptional subtype, and reduced patient survival [23]. We and others have demonstrated the preclinical therapeutic efficacy of IL13RA2‐targeted approaches using cell‐based platforms [24, 25, 26]. Recently, we reported the pharmacokinetic profile of the hBTE, demonstrating selective tumor accumulation, blood–tumor barrier (BTB) penetration via both passive diffusion and T cell–dependent mechanisms, and intratumoral persistence for over 24 h following systemic administration in mice bearing GBM xenografts [27].

Critical gaps remain in defining the key properties required for clinical translation of BTEs in solid tumors, particularly with respect to humanization and performance within the central nervous system. In this study, we describe the antibody's humanization strategy for selecting the lead hBTE molecule designed to target human IL13RA2 and CD3E, and evaluate its safety and therapeutic efficacy. We demonstrate robust, target‐specific antitumor activity in primary and recurrent GBM, brain metastases of breast cancer, and IL13RA2‐expressing extracranial tumors. These findings provide a preclinical foundation for the clinical translation of IL13RA2‐targeted hBTE therapy in GBM, brain metastases, and peripheral solid tumors.

## 2 Results

### 2.1 IL13RA2 is Highly Expressed in Malignant Gliomas

**FIGURE 1:**
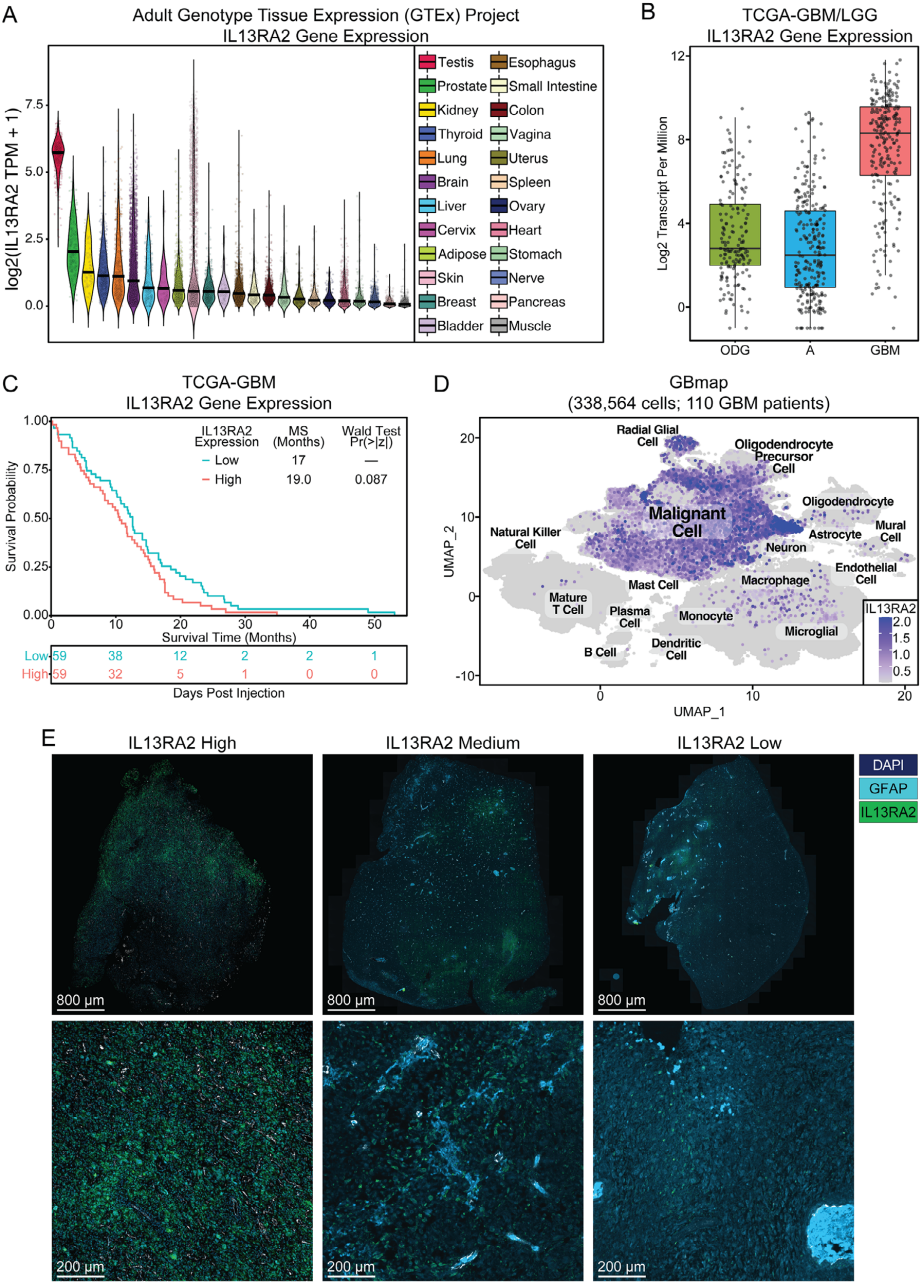
IL13RA2 is highly expressed in malignant gliomas. (A) IL13RA2 mRNA expression across 24 tissue types in the Adult Genotype Tissue Expression (GTEx) Project, showing low expression in all tissues except Testis. (B) IL13RA2 mRNA expression in the TCGA‐GBM/LGG dataset stratified by glioma genotype. A, IDH‐mutant Astrocytoma (n = 258); ODG, IDH‐mutant oligodendroglioma (n = 169); GBM, IDH‐WT Glioblastoma (n = 229). (C) Kaplan–Meier curves of overall survival in the TCGA‐GBM dataset based on IL13RA2 expression levels. High (Q3; n = 59) and low (Q1; n = 59) expression groups were defined using quantile cutoffs. Log‐rank test. (D) UMAP visualization of single‐cell RNA sequencing data from 110 GBM patients showing IL13RA2 expression predominantly restricted to malignant cells. (E) Multiplex sequential immunofluorescence (seqIF) of three GBM patient samples showing varying IL13RA2 protein expression. Scale bar, low magnification: 800 µm; high magnification: 200 µm.

IL13RA2 has been established as a glioma‐associated antigen that is absent in normal brain tissue and expressed at low levels in peripheral tissues, with the exception of the testes. Its expression has been associated with higher tumor grade, the mesenchymal transcriptional subtype, and reduced patient survival [20, 23]. Analysis of the Adult Genotype‐Tissue Expression project [28]. confirmed high IL13RA2 expression in the testes and minimal expression across major organs (Figure 1A). Consistently, analysis of the TCGA‐GBM/LGG dataset [29, 30] showed a steep increase in IL13RA2 mRNA expression from IDH‐mutant oligodendroglioma (n = 169) and astrocytoma (n = 258) to IDH‐wildtype GBM (n = 229), aligning with increasing tumor grade (Figure 1B). Survival analysis of IDH‐wildtype GBM patients with high and low IL13RA2 expressors in tumor tissue showed a trend toward worse survival in high expressors (Figure 1C). Analysis of a harmonized single‐cell RNA sequencing dataset from 110 GBM patients [31] confirmed that IL13RA2 expression is confined mainly to malignant cells (Figure 1D). As expected, multiplex sequential immunofluorescence (seqIF) of three GBM patient samples showed marked inter‐patient variability in IL13RA2 expression levels (Figure 1E).

### 2.2 Humanization of Anti‐IL13RA2 Antibody

**FIGURE 2:**
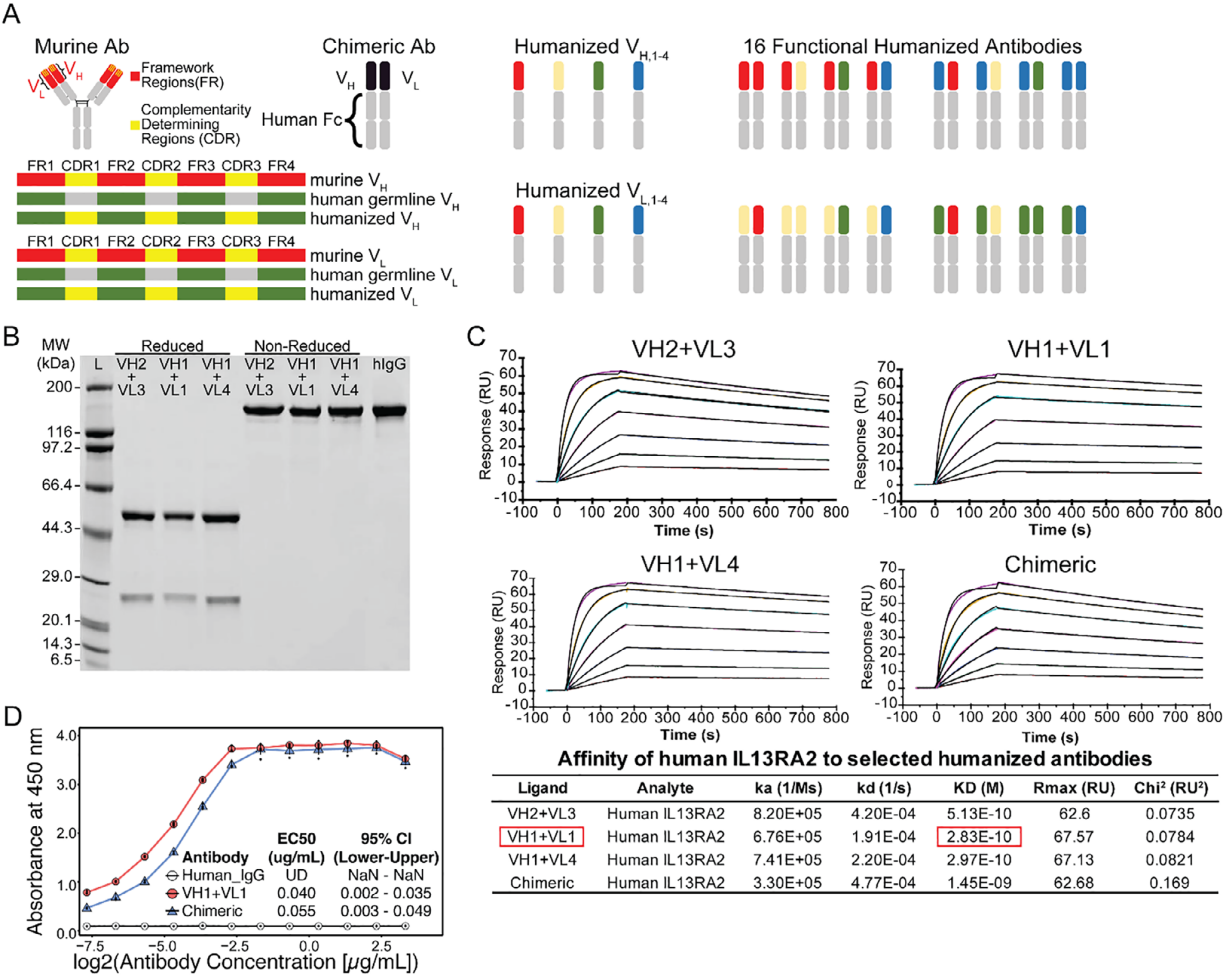
Humanization of anti‐IL13RA2 antibody. (A) Schematic of the humanization strategy. Murine CDRs were grafted onto four human VH and four human VL frameworks, generating a panel of 16 fully humanized antibody variants. (B) Western blot of selected humanized antibodies under reducing and non‐reducing conditions, showing high purity, correct assembly, and expected molecular weights. (C) Biacore T200 sensorgrams showing binding kinetics of selected humanized antibodies and the original chimeric antibody to human IL13RA2. The table summarizes kinetic parameters. (D) ELISA measuring binding of humanized VH1+VL1 antibody and chimeric antibody to human IL13RA2. EC50 values indicate comparable binding. UD, undetectable.

To enable clinical translation, the murine anti‐IL13RA2 antibody (clone 47) previously developed by our group [32] was humanized by grafting its complementarity‐determining regions (CDRs) onto four human IgG1 frameworks [33]. Gene synthesis was used to generate 16 full‐size antibody variants by combining four human variable heavy (VH) and four variable human light (VL) frameworks, each retaining the original murine CDRs (Figure 2A). SDS‐PAGE under reducing and non‐reducing conditions confirmed proper assembly and expected molecular weights of selected humanized antibody variants (Figure 2B). The affinity ranking of 16 humanized antibodies was performed, with three variants selected for further purification and affinity measurements compared with the murine chimeric antibody (clone 47). Notably, the humanized variants exhibited two to threefold higher affinity for IL13RA2 than the original chimeric antibody, and the VH1+VL1 variant was selected for further characterization due to its superior binding affinity (Figure 2C). ELISA confirmed comparable binding of the VH1+VL1 humanized antibody and the original chimeric antibody with similar EC50 values to hIL13RA2 (Figure 2D).

### 2.3 Humanized IL13RA2xCD3 Bispecific T Cell Engager Shows Potent and Antigen‐Dependent Activity Against GBM Cells

**FIGURE 3:**
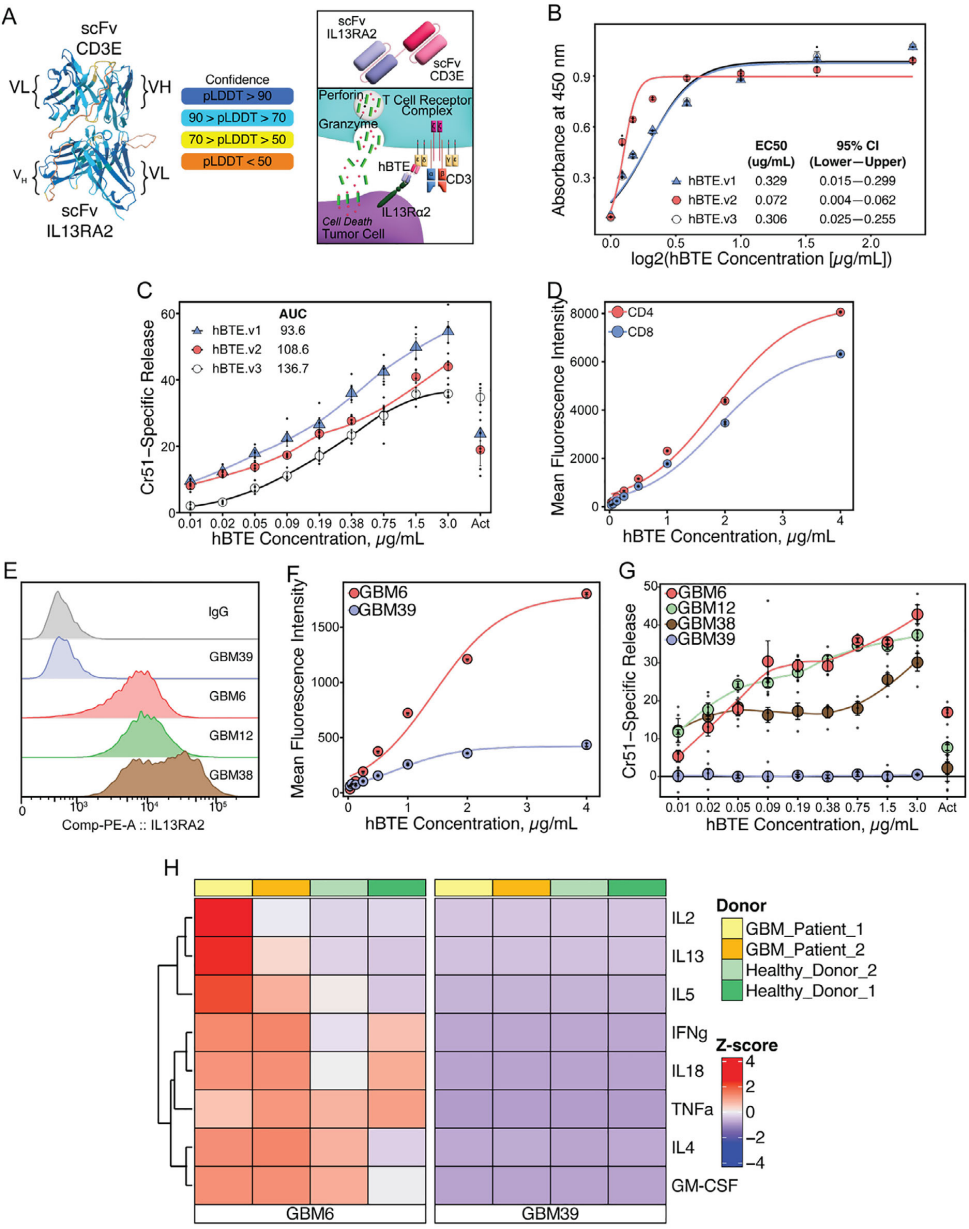
Humanized IL13RA2xCD3 bispecific T cell engager shows potent and antigen‐dependent activity against GBM cells. (A) AlphaFold‐predicted structure and schematic of humanized IL13RA2xCD3 bispecific T cell engager (hBTE), composed of anti‐IL13RA2 scFv, CD3E‐targeting scFv, and human Fc domain. (B) ELISA binding analysis of three hBTE scFv configurations to IL13RA2. (C) Chromium‐51 release assay showing dose‐dependent hBTE‐induced T cell–mediated killing of three hBTE scFv configurations, with hBTEv1 showing the strongest binding. (D) Flow cytometry mean fluorescent intensity showing dose‐dependent hBTE binding to CD4 and CD8 T cells using AF647‐conjugated anti‐His‐tag antibody. (E) Flow cytometric histogram of IL13RA2 surface expression on GBM6, GBM12, GBM39, and GBM39 PDX cell lines. (F) hBTE binding at varying concentrations to GBM6 and GBM39, assessed by flow cytometry and presented as mean fluorescent intensities. (G) Chromium‐51 release assay showing dose‐dependent hBTE‐induced T cell–mediated killing of GBM6, GBM12, and GBM38, but no effect on GBM39. (H) hBTE induced cytokine release in GBM6 co‐cultures with T cells from both healthy donors and GBM patients, whereas little to no cytokine production was observed in GBM39 co‐cultures, demonstrating antigen‐dependent activity.

Using a peptide linker to fuse a scFv from a fully human CD3E antibody previously tested in phase I clinical trials [34, 35] to the scFv derived from our VH1‐VL1 humanized IL13RA2 antibody, we engineered a CD3ExIL13RA2 bispecific T cell engager (hBTE) and evaluated its function against GBM cells. The AlphaFold‐predicted structure of the resulting molecule showed a hBTE with two scFvs that bridge CD3E on the T cell receptor complex to IL13RA2‐positive glioma cells, thereby activating T cells and inducing cell‐mediated cytotoxicity (Figure 3A).

To generate the most optimal hBTE, three different configurations of hBTE: hBTEv1 (CD3E V_H_V_L_‐V_L_V_H_ IL13RA2), hBTEv2 (CD3E V_H_V_L_‐V_H_V_L_ IL13RA2), and hBTEv3 (CD3E V_L_V_H_‐V_L_V_H_ IL13RA2), were generated and evaluated for binding to IL13RA2 and functionality. ELISA demonstrated comparable binding affinities across all hBTE configurations, with hBTEv2 showing the strongest binding to IL13RA2 (Figure 3B). In contrast, the chromium release cytotoxicity assay revealed that hBTEv1 most potently induced killing of IL13RA2‐positive GBM6 cells (Figure 3C). Based on these results, hBTEv1 was selected for subsequent studies and is hereafter referred to as hBTE.

Next, we used plate ELISA and flow cytometry to confirm the antigen specificity of hBTE. ELISA showed hBTE binding to IL13RA2 but not IL13RA1 or the BSA control (Figure S1A). Flow cytometric analysis of T cells incubated with hBTE and stained with an AF647‐conjugated anti‐His Tag antibody confirmed dose‐dependent binding to CD4+ and CD8+ T cells (Figure 3D; Figure S1B). To test hBTE functionality, we performed co‐culture experiments using four patient‐derived (PD) GBM cell lines and healthy donor T cells. Flow cytometry confirmed IL13RA2 surface expression on GBM6, GBM12, and GBM38, with GBM38 showing the highest expression density, but not GBM39 (Figure 3E). Consistent with this, hBTE is explicitly bound to IL13RA2‐positive GBM6 and not to IL13RA2‐negative GBM39 (Figure 3F; Figure S1C). A chromium release assay showed hBTE selectively induced T cell–mediated killing of IL13RA2‐positive GBM6, GBM12, and GBM38 cells, while no killing was observed in IL13RA2‐negative GBM39 (Figure 3G).

To further assess hBTE functionality, IL13RA2‐positive GBM6 or IL13RA2‐negative GBM39 cells were co‐cultured with T cells from healthy donors (n = 2) or GBM patients (n = 2), treated with hBTE, and analyzed for cytokine production using a multiplex Luminex assay. Of the cytokines evaluated, standard curves confirmed accurate detection of eight cytokines: IL‐2, IL‐4, IL‐13, IL‐5, IFN‐γ, IL‐18, TNF‐α, and GM‐CSF (Figure S1D). In GBM6 co‐cultures, hBTE induced cytokine release from healthy donor and GBM patients' T cells (Figure 3H). In contrast, little to no cytokine production was observed in GBM39 co‐cultures, validating that hBTE‐induced cytokine release is antigen dependent.

### 2.4 hBTE Significantly Extends the Survival of Mice Bearing a Patient‐Derived Xenograft of Primary and Recurrent GBM

To evaluate therapeutic efficacy, survival studies were performed using immunocompromised NSG MHCI/II DKO mice bearing orthotopic GBM xenografts derived from patientderived (PD) GBM cell lines. IL13RA2‐positive PD GBM cell lines were then stereotactically injected into the brain. Peripheral blood mononuclear cells (PBMCs) from healthy HLA‐matched donors were then adoptively transferred into mice 7 days after stereotactic tumor implantation. Male and female mice were randomized into treatment groups receiving hBTE (50 µg, i.p.), negative control BTE (NC‐BTE) containing mutations in the IL13RA2 scFv that abrogate IL13RA2 binding [25], or saline (i.p.) four times per week for 3 weeks and monitored for survival. Histochemical evaluation of brain sections from hBTE‐ and control‐treated animals across all PD‐GBM models was performed at endpoint and in long‐term surviving (LTS) mice as a secondary confirmation of tumor engraftment (Supplementary Figure S5A).

**FIGURE 4:**
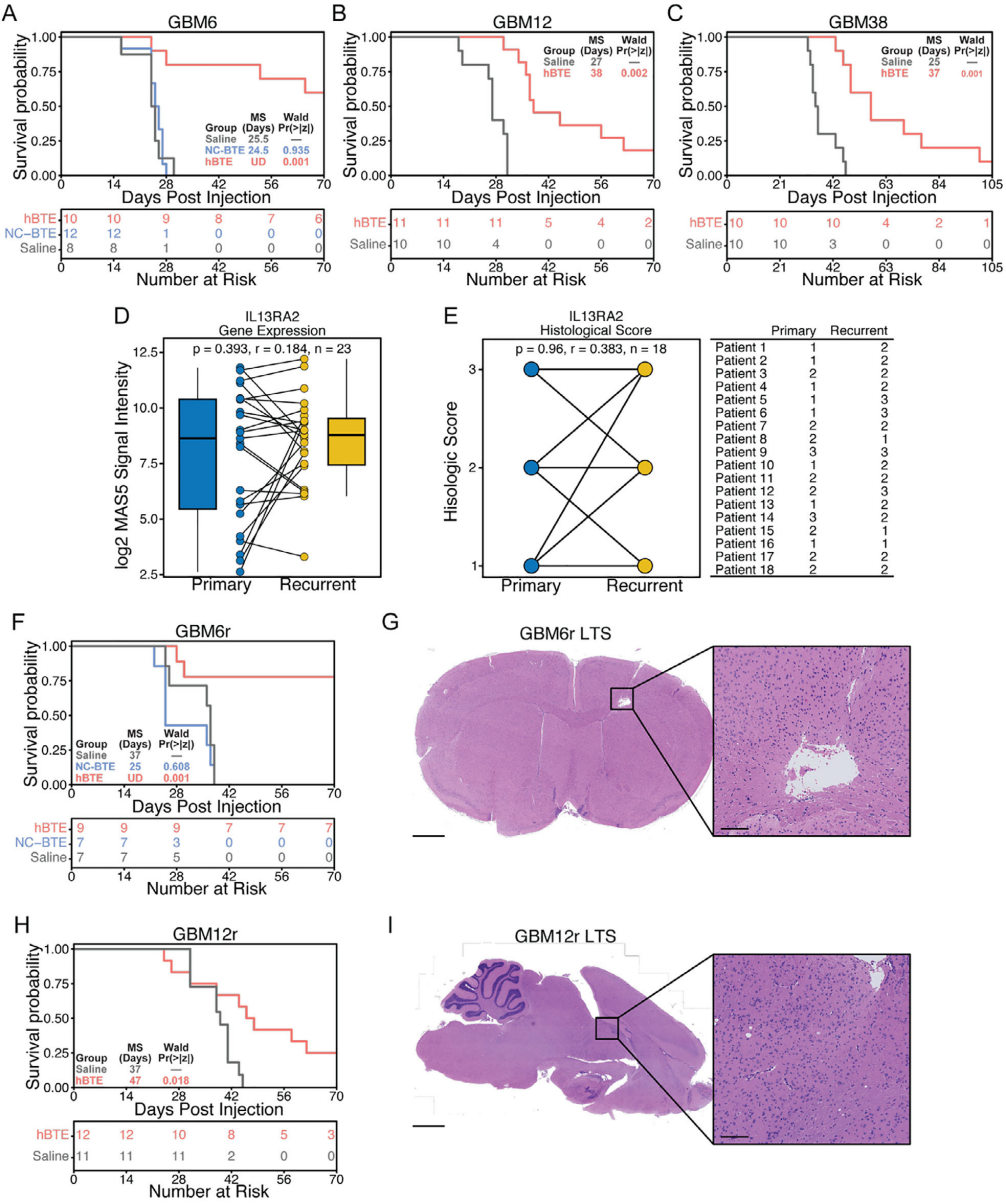
hBTE significantly extends the survival of patient‐derived xenograft models of primary and recurrent GBM. (A–C) Kaplan–Meier survival analyses of mice bearing primary GBM PDX tumors treated with hBTE, NC‐BTE, or saline. MS, median survival. (A) GBM6: hBTE (n = 10), NC‐BTE (n = 12), saline (n = 8). (B) GBM12: hBTE (n = 11), saline (n = 10). (C) GBM38: hBTE (n = 10), saline (n = 10). (D) Bulk RNA‐seq of matched GBM samples (GSE4271) comparing IL13RA2 between primary and recurrent tumors. Paired Wilcoxon signed‐rank test, one‐sided (Primary > Recurrent). n = 23. (E) Paired dot plot and summary table by patient with histological scoring of matched GBM samples showing comparable IL13RA2 protein expression in primary and recurrent tumors. Paired Wilcoxon signed‐rank test, one‐sided (Primary > Recurrent). n = 18. (F–I) Kaplan–Meier survival analyses and histological evaluation of mice bearing therapy‐resistant GBM PDX tumors treated with hBTE, NC‐BTE, or saline. (F) GBM6r model: hBTE (n = 9), NC‐BTE (n = 7), saline (n = 7). (G) H&E staining of brains from long‐term surviving GBM6r‐bearing mice treated with hBTE. (H) GBM12r model: hBTE (n = 12), saline (n = 11). (I) H&E staining of brains from long‐term surviving GBM12r‐bearing mice treated with hBTE. Survival statistical significance of Kaplan–Meier survival analyses was determined by the log‐rank test relative to saline controls.

In all three orthotopic GBM xenograft models, hBTE treatment significantly prolonged survival compared to controls (Figure 4A–C). In the GBM6 model, hBTE‐treated mice (n = 10; median overall survival [MS] = undefined; p = 0.001) showed a marked survival benefit compared to saline (n = 8; MS = 25.5 days) and NC‐BTE (n = 12; MS = 24.5 days) controls, with 60% of animals achieving long‐term survival (Figure 4A). Similarly, in the GBM12 model, hBTE treatment (n = 11; MS = 38 days; p = 0.002) extended survival relative to saline‐treated controls (n = 10; MS = 27 days), with 18% of animals reaching long‐term survivor status (Figure 4B). In the GBM38 model, hBTE also significantly extended survival (n = 10; MS = 37 days; p = 0.001) compared to saline‐treated controls (n = 10; MS = 27 days), with one animal classified as a long‐term survivor (Figure 4C); Given the reduced magnitude of survival benefit in GBM38 model compared to GBM6 and GBM12, subsequent staining for IL13RA2 in brain sections collected at endpoint was performed to evaluate antigen escape. Results showed IL13RA2 expression was retained in both hBTE‐treated and saline control mice (Figure S5B).

Given the high recurrence of GBM, evaluating therapeutic efficacy in recurrent models is critical for informing experimental trial design. Recurrent GBM displays distinct molecular characteristics compared to the primary disease, marked by unique mutation profiles, phenotypic plasticity, and epigenetic reprogramming that collectively drive resistance to standard‐of‐care (SOC) therapies [36, 37, 38, 39, 40]. Analysis of bulk RNA sequencing from 23 matched primary and recurrent GBM samples (GSE4271) showed that, despite interpatient variability, IL13RA2 expression was maintained in recurrent tumors (Figure 4D). Similarly, histological scoring showed no significant difference in IL13RA2 protein expression across 16 matched primary and recurrent GBM tumors (Figure 4E), validating that expression of IL13RA2 was largely retained in recurrent tumors. We previously established therapy‐resistant PD GBM xenograft models, generating them by orthotopic implantation of treatment naïve tumors into athymic nude mice followed by repeated exposure to SOC therapies, including fractionated radiation (XRT) and temozolomide (TMZ) [41]. Two of these resistant GBM xenograft models, GBM6 m3378 (GBM6r) and GBM12 m2671(GBM12r), respectively derived from the treatment naïve GBM6 and GBM12 treatment naïve PD cell lines, were used to evaluate hBTE in a recurrent setting. Chromium release assays demonstrated that hBTE selectively promoted T cell–dependent cytotoxicity against GBM6r (Figure S2A) and GBM12r (Figure S2B) cells. In vivo, hBTE treatment significantly prolonged survival in GBM6r‐bearing mice (n = 9; MS = undefined; p = 0.001) over saline (n = 7; median = 37 days) and NC‐BTE (n = 7; median = 25 days) controls, with 78% achieving long‐term survival (Figure 4F). Histological analysis of long‐term survivors showed no evidence of residual tumor (Figure 4G). Likewise, hBTE‐treated GBM12r‐bearing mice (n = 12; MS = 47 days; p = 0.018) exhibited extended survival relative to saline controls (n = 11; median = 39 days), with 25% reaching long‐term survival (Figure 4H). Histological assessment of these long‐term survivors confirmed an absence of detectable tumor (Figure 4I).

### 2.5 hBTE Enhances T Cell Infiltration and Improves Outcomes in Models of Brain Metastases and Subcutaneous Extracranial Tumors

**FIGURE 5:**
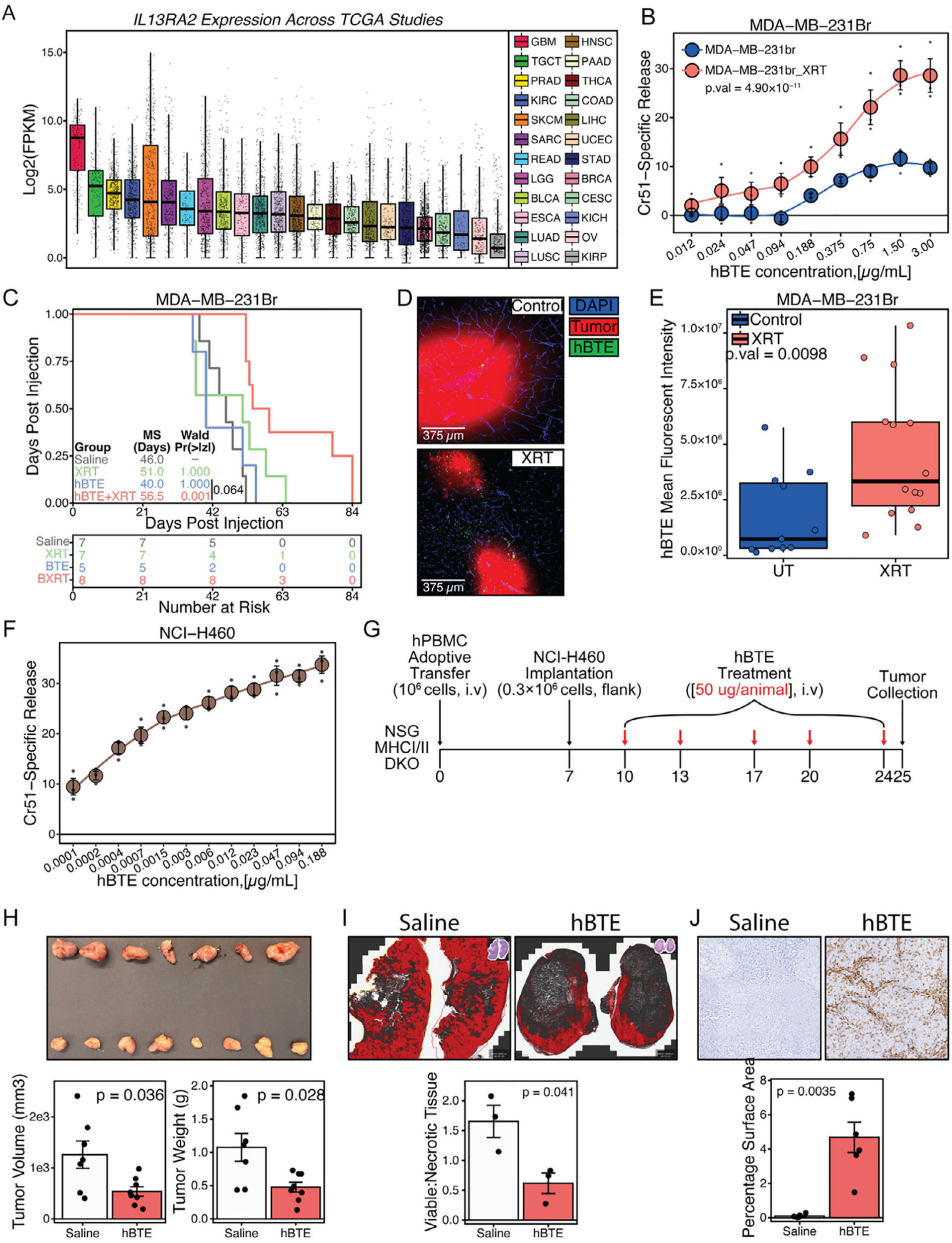
hBTE enhances T cell infiltration and improves outcomes in models of brain metastases and subcutaneous extracranial tumors. (A) IL13RA2 mRNA expression across 25 tumor types in the TCGA Pan‐Cancer dataset, showing variable, patient‐specific expression. (B) Chromium release assay showing dose‐dependent hBTE‐induced T cell–mediated killing of MDA‐MB‐231Br cells treated with or without XRT. Two‐way ANOVA (factors: XRT‐treatment and hBTE Concentration); p‐value corresponds to the main effect of hBTE Concentration. (C) Survival curves of nude mice bearing intracranial MDA‐MB‐231Br tumors treated with hBTE (50 µg, i.v., twice weekly), radiotherapy (XRT; 5 × 2 Gy), or both. MS, median survival. Statistical significance was determined by the log‐rank test relative to the saline control. (D) Representative immunofluorescent images of brain sections from control and XRT‐treated mice following hBTE administration, showing hBTE signal distribution. Blue, DAPI; red, tumor; green, hBTE. Scale bar, 375 µm. (E) Quantification of hBTE fluorescence intensity in XRT‐treated mice compared to controls. Statistical significance was determined by Student's t‐test. (F) Chromium release assay showing dose‐dependent hBTE‐mediated killing of NCI‐H460 lung cancer cells. (G) Schematic of the in vivo experimental design: human PBMCs were adoptively transferred into NSG MHC I/II DKO mice, followed by subcutaneous implantation of NCI‐H460 tumors and hBTE treatment. (H) Quantification of tumor burden in mice treated with hBTE (n = 8) or saline (n = 7) was measured as tumor weight and volume. Unpaired Welch's t‐test. (I) Histological analysis of tumors showing significantly increased tumor cell death in hBTE‐treated mice, assessed by viable‐to‐necrotic tissue ratio. (J) Quantification of intratumoral CD3+ T cells, showing significantly increased infiltration in hBTE‐treated tumors. Unpaired Welch's t‐test.

In addition to being a well‐characterized glioma‐associated antigen, IL13RA2 is also expressed in several extracranial malignancies [42]. Analysis of the TCGA Pan‐Cancer dataset showed detectable, patient‐specific IL13RA2 mRNA expression with variable levels across 25 tumor types (Figure 5A; abbreviations in Table S1). Thus, we evaluated hBTE function against triple‐negative breast cancer (TNBC) brain metastatic and extracranial lung cancer cell lines.

Flow cytometry confirmed moderate IL13RA2 surface expression on MDA‐MB‐231Br, a TNBC brain metastatic cell line, with 55.9% of live cells staining positive (Figure S2C). In a chromium release assay, hBTE induced dose‐dependent T cell–mediated killing of MDA‐MB‐231Br cells (Figure 5B) with higher responses recorded with cells treated with a single 4Gy dose of irradiation (XRT). To test hBTE activity in vivo, MDA‐MB‐231Br cells were intracranially injected into female athymic nude mice, infused with the donor's PBMCs, injected hIL2 biweekly, and treated with either hBTE (50 µg per dose, i.v.) or saline twice weekly for 3 weeks. Despite demonstrating in vitro activity, hBTE monotherapy did not improve survival in MDA‐MB‐231Br–bearing mice (Figure S2D), potentially reflecting the limited penetration of hBTE into the brain metastatic niche. Given that radiation therapy (XRT) can enhance blood–tumor barrier (BTB) permeability through vascular remodeling [43, 44, 45], an additional experiment was performed in mice treated with hBTE (50 µg per dose, i.v., twice weekly), alone or in combination with fractionated XRT (5 × 2 Gy, total 10 Gy). Neither hBTE (MS: 40.0 days) nor XRT (MS: 51.0 days) significantly extended survival compared to saline controls (MS: 46.0 days) (Figure 5C). However, sequential treatment with XRT followed by hBTE significantly prolonged survival (MS: 56.5 days; p = 0.001 vs. control) (Figure 5C) of mice. To understand why hBTE extended survival when coupled with XRT, we quantified the signal for Cy5‐labeled hBTE in the brains of control and XRT mice (Figure 5D; single channel images in Figure S2E). Compared to control, hBTE signal intensity was significantly higher in XRT‐treated animals with apparent accumulation of hBTE in peritumoral space (p = 0.0098) (Figure 5E).

To assess efficacy in solid tumors outside the CNS, we next tested hBTE activity in the IL13RA2‐expressing lung cancer cell line NCI‐H460. Flow cytometry confirmed high IL13RA2 surface expression in NCI‐H460, with 73.5% of live cells staining positive (Figure S2F). The chromium release assay revealed that hBTE induced T cell–mediated killing of NCI‐H460 in a dose‐dependent manner (Figure 5F). To evaluate in vivo, human PBMCs (1 × 10^7^ cells, i.v.) were adoptively transferred into NSG MHC I/II DKO mice, followed 7 days later by subcutaneous injection of NCI‐H460 cells (3 × 10^5^) into the flank. 3 days later, hBTE treatment (50 µg per dose, i.v., twice weekly) for about 2 weeks, after which tumors were harvested and analyzed (Figure 5G). hBTE (n = 8) treated animals had significantly reduced tumor size, both by volume (p = 0.036) and weight (p = 0.028), compared to saline controls (n = 7) (Figure 5H). Histological analysis revealed that hBTE‐treated tumors had significantly increased tumor cell death, indicated by a reduced viable‐to‐necrotic tissue ratio compared to saline‐treated controls (p = 0.041) (Figure 5I; H&E in Figure S2G). This was accompanied by robust T cell infiltration in hBTE‐treated tumors (p = 0.0035) (Figure 5J; full tissue image in Figure S2H). To confirm that this response was specific to hBTE and not attributable to off‐target immune stimulation, procedural effects associated with injection, or other unrelated variables, we repeated the experiment using saline‐ and NC‐BTE–treated animals. We observed no differences in tumor volume or weight between NC‐BTE and saline controls (Figure S2I).

### 2.6 hBTE Exhibits No Off‐Target or Systemic Toxicities From BTEs in Tumor‐Bearing Mice

**FIGURE 6:**
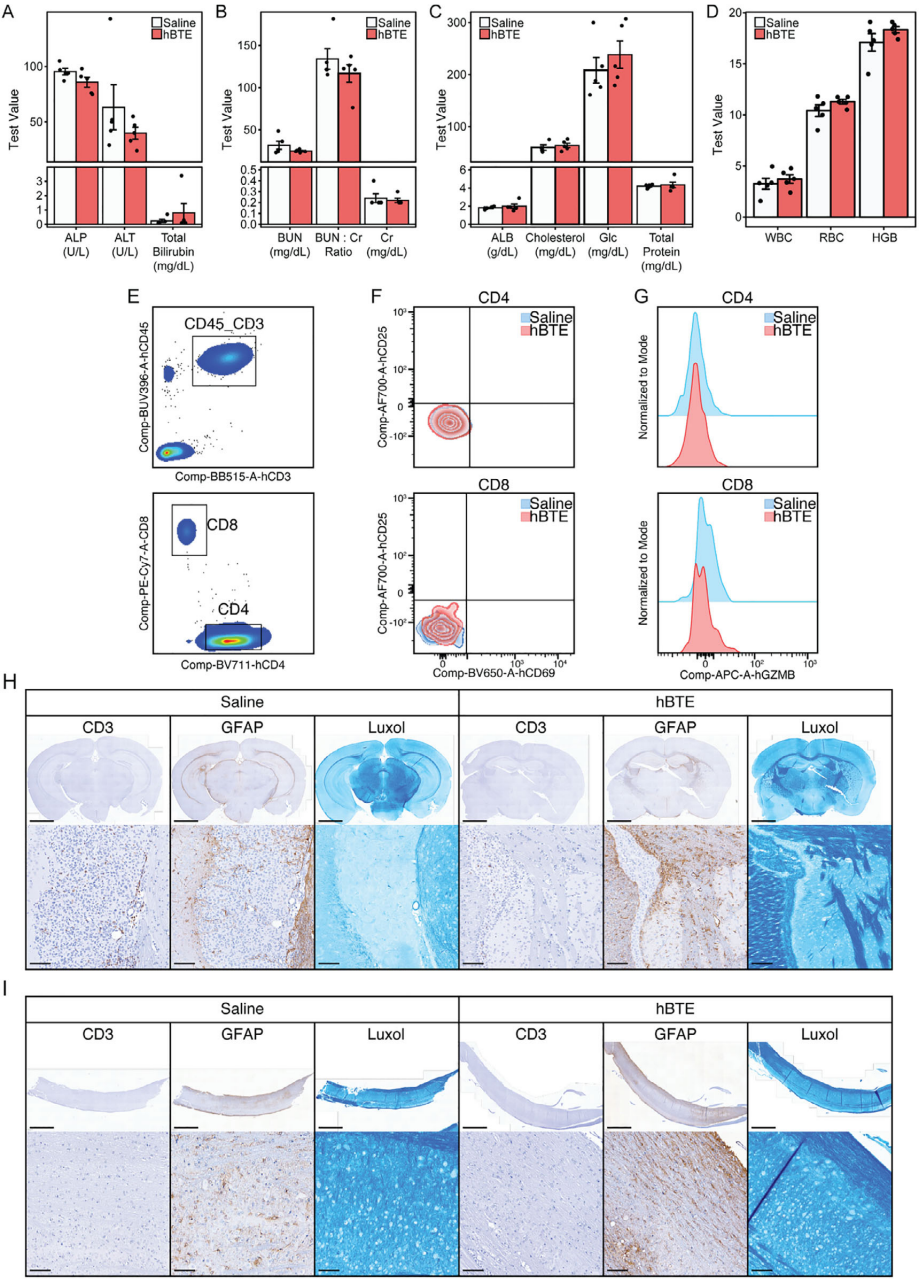
hBTE exhibits no systemic toxicities from hBTEs in tumor‐bearing mice. (A–I) Evaluation of systemic and CNS toxicity following repeated hBTE administration in GBM6 PDX–bearing mice. (A) Liver function parameters, including alkaline phosphatase (ALP), alanine aminotransferase (ALT), and total bilirubin, showed no significant differences between hBTE‐ and saline‐treated controls. (B) Kidney function parameters, including blood urea nitrogen (BUN), BUN: creatinine (Cr) ratio, and creatinine levels, showed no significant differences between groups. Metabolic parameters, including albumin (ALB), cholesterol, glucose (Glc), and total protein, showed no changes between treatment groups. (D) Hematologic analysis showing comparable WBC, RBC, and HGB counts between treatment groups. (E) Flow cytometric analysis of peripheral human T cells showing no significant differences in (F) CD25^+^CD69^+^ double‐positive frequencies or (G) GZMB expression in CD8^+^ and CD4^+^ T cells between hBTE‐ and saline‐treated mice. (H) Brain histology showing similar CD3^+^ T cell abundance and preserved myelin structure by Luxol Fast Blue staining, indicating absence of neurotoxicity or demyelination. (I) Spinal cord sections showing no CD3^+^ T cell infiltration and normal myelin and astrocyte morphology by Luxol Fast Blue and GFAP staining, indicating lack of neuroinflammation or CNS toxicity.

Comprehensive preclinical assessments of systemic, off‐target, and on‐target/off‐tumor toxicities of biologics are essential to ensuring safe clinical translation. Accordingly, we assessed potential hBTE‐associated toxicities in vivo in GBM6‐bearing mice following repeated dosing. A veterinary clinical chemistry panel was used to evaluate liver, kidney, and metabolic function, as well as hematologic parameters. No significant differences were observed in alkaline phosphatase (ALP), alanine aminotransferase (ALT), or total bilirubin between hBTE‐ and saline‐treated controls (Figure 6A). Similarly, blood urea nitrogen (BUN), BUN:creatinine (Cr) ratio, and Cr levels were comparable between groups (Figure 6B). No changes were detected in albumin (ALB), cholesterol, glucose (Glu), or total protein (Figure 6C). Last, white blood cell (WBC), red blood cell (RBC), and hemoglobin (HGB) counts remained unchanged (Figure 6D). These results indicate that hBTE treatment did not elicit detectable liver, kidney, metabolic, or hematologic toxicities. Statistical analyses for all parameters are provided in Table S2.

To assess whether hBTE induces peripheral T cell activation consistent with cytokine release syndrome (CRS), flow cytometry was used to measure activation markers in donor T cells isolated from mouse blood (Figure 6E). In both CD8^+^ and CD4^+^ T cells, no differences in the frequency of CD25^+^CD69^+^ double‐positive cells were observed between hBTE‐ and saline‐treated controls (Figure 6F). Similarly, GZMB expression in CD8^+^ and CD4^+^ T cells showed no difference between groups (Figure 6G).

We collected all major organs for histopathological analysis to assess potential tissue damage comprehensively. In the CNS, CD3^+^ T cell abundance was comparable between hBTE‐ and saline‐treated animals (Figure 6H; H&E in Figure S2A). Luxol Fast Blue staining showed similar myelin composition across groups, with no evidence of hBTE‐induced neurotoxicity or demyelination (Figure 6H). In the spinal cord, no CD3^+^ T cell infiltration was observed in either group, and both Luxol Fast Blue and GFAP staining revealed normal myelin structure and astrocyte morphology, indicating that hBTE does not elicit neuroinflammation or CNS toxicity (Figure 6I; H&E in Figure S2B). Similarly, no signs of T cell infiltration in response to hBTE treatment and toxicity were observed in all peripheral organs evaluated in the study (Figure S3C–H).

To further assess potential on‐target/off‐tumor toxicities, we performed a chromium‐51 release assay using two normal human fibroblast cells (NHFC), isolated from healthy donors, as target cells. Despite having expressed IL13RA2 (Figure S3I), hBTE did not induce T cell–mediated killing of IL13RA2‐positive human fibroblast cells in either donor (Figure S3J). To assess potential off‐target toxicities, IL13RA2‐negative human renal cells (HEK293T) and normal human keratinocytes (NHKC) were incubated with hBTE and stained with an AF647‐conjugated anti‐His Tag antibody. The binding of hBTE was not detected in either HEK293T or NHKC, but the robust binding was evident in IL13RA2‐positive GBM6 cells (positive control) (Figure S5C,D).

## 3 Discussion

The primary objective of this study was to validate our fully humanized BTE lead candidate in GBM by confirming its antigen‐specific activity across multiple GBM xenograft models following systemic administration and demonstrating its favorable safety profile. These findings establish a strong preclinical foundation for advancing hBTE therapy toward clinical evaluation in IL13RA2‐positive GBM. Given IL13RA2 expression across multiple solid tumor types, extending hBTE testing to additional IL13RA2‐expressing cancers may further expand its therapeutic scope. For brain metastases and peripheral tumors, overcoming the limitations of BBB and BTB penetration will be an important next step, requiring optimization of delivery routes and integration with complementary strategies such as XRT or focused ultrasound. Altogether, this work supports the clinical translation of hBTE in GBM and continued evaluation in other IL13RA2‐expressing malignancies, to deliver effective therapies for patients with treatment‐refractory disease.

Bispecific T‐cell engagers (BTEs) represent a rapidly expanding class of targeted immunotherapies with proven efficacy in hematologic malignancies, though their application in solid and CNS tumors remains emerging. Penetration across the BBB, immunosuppressive microenvironment, and heterogeneous antigen expression are among the limitations of targeted therapy in CNS tumors. IL13RA2 is a tumor‐associated antigen enriched in glioma [22]. Its expression increases with tumor grade and correlates with poor patient outcomes. Although expression is heterogeneous across and within patients, single‐cell RNA sequencing reveals that IL13RA2 is predominantly expressed by glioma cells in GBM. In contrast, its expression is low to undetectable in all major organs except the testes, and IL13RA2 is also expressed in peripheral solid tumors and brain metastases. These features make IL13RA2 an attractive target for the development of IL13RA2‐directed therapies [24, 46].

This study used our previously developed and validated high‐affinity murine anti‐IL13RA2 antibody (clone 47) [27, 32, 47]. as a starting point for humanization for clinical translation, aiming to reduce immunogenicity, improve pharmacokinetics, and minimize the risk of human anti‐mouse antibody (HAMA) responses that could accelerate clearance in patients [48, 49]. The scFv from this humanized antibody was then fused with a fully human anti‐CD3 scFv [34]. to generate three distinct configurations of a fully humanized bispecific T‐cell engager (hBTE), each differing in the VH–VL orientation of the scFv domains. The configuration of a BTE, including the order of the scFvs and the orientation of the variable heavy and light domains within each scFv, can influence its functional properties. These structural features may affect expression, stability, antigen binding, and the geometry of T cell engagement [50, 51, 52, 53]. For example, prior studies have shown that altering VH–VL orientation can modulate activity in HER2 × CD3, EGFR × CD3, and CD19 × CD3 bispecific constructs [50, 54, 55]. In our study, the CD3E VHVL–VHVL–IL13RA2 configuration exhibited the highest IL13RA2 binding by ELISA. Although hBTEv1 and hBTEv3 demonstrated comparable binding affinity, the CD3E VHVL–VLVH IL13RA2 configuration showed the strongest T cell–mediated cytotoxicity in glioma models. These results indicate that functional activity is not determined by binding affinity alone and may be influenced by domain orientation. Based on its overall functional performance, hBTEv1 was selected for further evaluation.

We demonstrated that hBTE mediates robust antigen‐specific activation, cytokine release, and T cell‐mediated killing of IL13RA2‐positive PD GBM cells. In vivo, this translated into a significant survival benefit across all three IL13RA2‐expressing GBM xenograft models of primary disease (GBM6, GBM12, and GBM38). The reduced magnitude of survival benefit in the GBM38 model was unexpected, given that GBM38 exhibited the highest IL13RA2 expression by flow cytometric analysis. As antigen escape remains a common observation in targeted therapies, we evaluated IL13RA2 expression in brain sections from both hBTE‐treated and saline control mice at endpoint. IL13RA2 expression was retained in both hBTE and saline‐treated mice, suggesting that resistance in GBM38 was likely driven by mechanisms distinct from antigen escape. GBM, despite multimodality initial therapy, almost invariably recurs. Although genomically recurrent tumors retain the features of the initially resected tumors, some recurrent tumors may exhibit distinct molecular features compared to the primary disease, including elevated tumor mutational burden (TMB), unique mutational landscapes, phenotypic plasticity, and epigenetic reprogramming that collectively drive resistance to standard‐of‐care (SOC) therapies [36, 37, 38, 39, 40]. In the recurrent setting, there is no well‐established or effective standard of care; therefore, participation in clinical trials is commonly recommended. This underscores the dire need for better treatment options for patients with both newly diagnosed and recurrent GBM, and evaluating therapeutic efficacy in recurrent models is essential for guiding experimental trial design. Analysis of two independent patient‐matched datasets demonstrated that IL13RA2 expression is maintained at recurrence at both RNA and protein levels. To test hBTE efficacy in the recurrent setting, we utilized two previously established therapy‐resistant PD GBM xenograft models, GBM6 m3378 (GBM6r) and GBM12 m2671 (GBM12r), derived from their respective treatment‐naïve GBM6 and GBM12 counterparts [41]. Both models exhibit increased TMZ resistance and elevated tumor mutational burden (TMB). These models also acquired mutations in DNA mismatch repair genes, with GBM6r carrying alterations in MSH6 and PMS2, and GBM12r harboring a mutation in MSH6 [41]. Nevertheless, hBTE treatment again showed robust antigen‐specific T cell‐mediated killing in vitro and significantly extended survival in both GBM6r and GBM12r models in vivo. These findings indicate that hBTE displays potent activity in both primary and therapy‐resistant GBM and emphasize its potential clinical relevance for patients with IL13RA2‐expressing tumors.

IL13RA2 is commonly overexpressed in glioblastoma but is also detected in several other solid tumors, including those associated with brain metastases. The blood–tumor barrier (BTB) in brain metastases differs structurally and functionally from that in GBM. Comparative imaging studies have demonstrated the restrictive nature of the BTB in brain metastases of breast cancer [45] and other solid tumors [56, 57, 58, 59]. Not surprisingly, hBTE monotherapy did not improve outcomes in the breast cancer brain metastasis (BCBM) model, likely due to inadequate penetration of the therapeutic across the BTB. It is well established that radiation therapy (XRT) transiently increases BTB permeability in BCBM through radiation‐induced alterations in vascular dynamics [43, 44, 45]. Consistent with this, our group previously showed that an anti‐HER2 nanobody exhibited limited penetration in HER2^+^ BCBM models [60]. In this study, neoadjuvant XRT significantly enhanced survival in hBTE‐treated mice, and confocal microscopy demonstrated increased peritumoral accumulation of the hBTE in BCBM. Our in vitro data demonstrated that BM cells treated with a single dose of XRT responded better to T‐cell cytotoxicity in the presence of hBTE than controls, suggesting that sensitization of BCBM cells might also have improved BTE‐mediated tumor responses to T cells in vivo. These results indicate that optimizing BTE delivery through XRT‐induced BTB disruption may be critical for effectively treating brain metastases. Accordingly, local or intrathecal administration of T cells and BTEs that bypass the BTB should thus be explored in the follow‐up study.

As a representative of peripheral solid tumors, lung cancer was evaluated in subcutaneous xenograft models, where hBTE treatment demonstrated an apparent therapeutic activity. Together with recent reports of BTE efficacy in melanoma [61], our findings support the broader potential of BTEs as a therapeutic strategy for IL13RA2‐expressing peripheral cancers. Thus, further evaluation of hBTE in IL13RA2‐expressing cancers is warranted, and future studies should rigorously assess its efficacy in additional peripheral cancer models in situ.

Cytokine release syndrome (CRS) is a serious immune‐mediated toxicity caused by excessive T cell activation. Cytokines released by activated T cells drive systemic immune cell activation and cytokine amplification, leading to widespread inflammation that can result in organ failure and death. CRS is a major safety concern in cancer immunotherapy, with adverse effects documented following immune checkpoint blockade, CAR T cell, and BTE therapy [62, 63, 64, 65, 66]. In addition to CRS, BTE treatment has also been associated with neutropenia and infection‐related complications [67]. In particular, constructs incorporating scFvs connected by flexible linkers, such as blinatumomab, have been linked to an increased incidence of cytokine release syndrome, neurotoxicity, and hematologic toxicity, including immune effector cell–associated neurotoxicity syndrome (ICANS) and cytopenias [68]. Our recent study in immunocompetent hosts shows that a fully murine BTE containing an scFv recognizing mouse CD3E induces T cell activation confined to the tumor microenvironment, occurring only upon engagement with IL13RA2‐expressing cancer cells [47]. In this study, hBTE elicited robust in vitro T cell‐mediated killing of IL13RA2‐expressing GBM cells without inducing cytotoxicity in IL13RA2‐positive fibroblast cells. In vivo, hBTE‐treated humanized mice bearing PD GBM6 gliomas showed no evidence of hematologic toxicity, neurotoxicity, including demyelination, or systemic inflammation. These findings indicate that hBTE treatment elicits potent, antigen‐restricted T cell activity within the tumor while minimizing systemic immune activation and associated toxicities. However, murine and humanized mouse models are inherently limited by species‐specific antigen expression and incomplete representation of human tissue distribution. Although these models offer initial safety insights, definitive evaluation of hBTE‐associated off‐target and on‐ target off‐tumor toxicities will require clinical studies.

Few BTE molecules have been evaluated in preclinical glioma models or in clinical trials outside of trispecific, cell‐ or virus‐delivered, and T‐cell precomplexed formats. These include an EGFRvIII × CD3 [69] and a B7‐H3 × CD3 BTEs [70]. Furthermore, no BTEs targeting IL13RA2 have entered clinical testing for GBM. The only active clinical effort in this area is a Phase 1 trial scheduled to begin in December 2026, evaluating BRiTE, a bispecific hEGFRvIII × CD3 construct in patients with WHO grade 4 glioma (NCT04903795). The previous EGFRvIII‐targeting BiTE trial, AMG 596 (NCT03296696), was terminated for business and strategic prioritization reasons. Therefore, there remains a critical need to expand the evaluation of novel single‐chain variable fragments as therapeutic strategies for central nervous system malignancies.

In summary, this work establishes hBTE as a promising therapeutic candidate for IL13RA2‐expressing GBM. These findings provide a strong foundation for clinical translation and potential evaluation in other IL13RA2‐expressing solid tumors.

## 4 Experimental Section

### 4.1 Cell Lines and Cell Culture

Patient‐derived (PD) GBM lines GBM6, GBM12, GBM38, and GBM39 were obtained from the Mayo Clinic Brain Tumor Patient‐Derived Xenograft National Resource (Mayo Clinic, Rochester, MN, USA) [71]. Temozolomide‐resistant GBM6r (GBM6 m3378) and GBM12r (GBM12 m2671) cell lines were generated as described [41]. All PD GBM lines were maintained through serial flank passaging and cultured for no more than three passages before use. Brain metastatic breast cancer cell line MDA‐MB‐231Br3 (RRID: CVCL_A0YY) was obtained from Dihua Yu (MD Anderson Cancer Center, Houston, TX, USA) [72, 73]. The NCI‐H460 (RRID: CVCL_0459) lung carcinoma cell line was obtained from NIH [74, 75]. PD GBM lines and MDA‐MB‐231Br cells were grown in Dulbecco's Modified Eagle Medium (Corning Life Sciences; Corning, New York, USA; Cat. 10‐013‐CV) supplemented with 10% v/v fetal bovine serum (FBS) (R&D Systems; Minneapolis, Minnesota, USA; Cat. No. S11550) and 1% v/v Penicillin‐Streptomycin (Gibco; Cat. 15140‐122). HEK293T (RRID: CVCL_0063) were grown in Dulbecco's Modified Eagle Medium supplemented with 10% v/v FBS. NCI‐H460 lung carcinoma cell line was grown in RPMI 1640 media (Corning Life Sciences; Cat. 10‐040‐CM) supplemented with 10% v/v FBS and 1% v/v Penicillin‐Streptomycin. All cell lines tested negative for Mycoplasma contamination prior to experimental use. Normal human fibroblast cells (NHLC) were dissociated from lung tissues collected from deceased donors and utilized under a secondary use protocol (University of Pennsylvania, Philadelphia, PA) [76]. Normal human keratinocyte cells (NHKC) were obtained from the Skin Biology & Diseases Resource‐Based Center (Northwestern University, Chicago, IL) under IRB STU00009443‐CR0009. Details of all cell lines, including source, RRID, and citation, are provided in Table S5.

### 4.2 Generation of Humanized IL13RA2‐Targeting Antibody

The variable regions (CDR) of the heavy (VH) and light (VL) chains of the murine monoclonal antibody IL13RA2 (clone 47) were previously reported by our group [32]. The humanization of clone 47 was performed by GeneScript NJ. The VH and VL CDRs were grafted onto four human germline framework sequences selected based on high sequence identity using the IgBlast database, resulting in four humanized VL, denoted as VL1, VL2, VL3, and VL4, and four VH, denoted as VH1, VH2, VH3, and VH4. Each chain was fused with a human IgG1 Fc sequence to mediate affinity measurements and purification using protein A Sepharose. Chimeric antibody comprised of murine VH and VL derived from sequences of the IL13RA2 (clone 47) antibody and human IgG1 Fc fragment served as a control in the antibody affinity measurements. The DNA sequences encoding the chimeric antibody heavy and light chains were synthesized and inserted into the pCDNA3.4 vector to construct expression plasmids of full‐length IgGs. Expression of the chimeric antibody was conducted in Expi293F cell culture, and the supernatants were purified using an affinity purification column. The purified antibody was buffer‐exchanged into PBS using a dialysis bag. The concentration and purity of the purified protein were determined by OD280 and SDS‐PAGE, respectively.

For affinity ranking, antibodies were immobilized on the sensor chip through the Fc capture method. Human IL13RA2 was used as the analyte. The surface was regenerated before the injection of another antibody. The process was repeated until all antibodies were analyzed. The off‐rates of antibodies were obtained by fitting the experimental data locally to a 1:1 interaction model using the Biacore 8K evaluation software. The antibodies were ranked by their dissociation rate constants (off‐rates, *kd*). Based on the ranking result, the top 3 clones were selected. The top 3 binders were chosen to express in Expi293F cell culture. The recombinant IgGs secreted to the medium were purified using resin A affinity chromatography. The affinity of purified antibodies binding to Human IL13RA2 was individually determined using Biacore T200. Antibodies were immobilized on the sensor chip through the Fc capture method. Human IL13RA2 was used as the analyte. The data of dissociation (kd) and association (ka) rate constants were obtained using Biacore T200 evaluation software. The equilibrium dissociation constants (KD) were calculated from the ratio of kd over ka. The VH1/VL1 paired humanized antibody was used to design the hBTE.

### 4.3 Generation of hBTE

We use the term “BTE” to refer to bispecific T‐cell engager molecules as a class of engineered antibody therapeutics, and “hBTE” as the abbreviation for our humanized anti‐IL13RA2 BTE investigated in this study. This is not to be confused from Amgen's BiTE platform [77].

A previously reported BTE construct design served as the template for developing the humanized bispecific T cell engager (hBTE), with modifications introduced to enhance clinical translatability [25]. The IL13RA2‐targeting domain consisted of a fully humanized single‐chain variable fragment (scFv) derived from the VH1/VL1 paired antibody generated in this study, replacing the murine IL13RA2‐targeting scFv (mAb47) [32]. The CD3E‐targeting domain was substituted with a fully human anti‐CD3 scFv (28F11‐AE, commercial name Foralumab) [34]. The two scFvs were connected via a 23‐amino acid flexible Gly‐Ser (GlyS) linker.

To generate the most effective hBTE, the anti‐CD3 and anti‐IL13RA2 scFvs were assembled in three distinct configurations: hBTEv1 (scFvCD3E VHVL–linker–scFv VLVH IL13RA2), hBTEv2 (scFv CD3E VHVL–linker– scFv VHVL IL13RA2), and hBTEv3 (scFv CD3E VLVH–linker– scFv VLVH IL13RA2). These variants were subsequently evaluated for IL13RA2 binding affinity and T cell–mediated cytotoxic activity.

### 4.4 Enzyme‐Linked Immunosorbent Assay

hBTE binding to human IL13Rɑ2 was assessed using an enzyme‐linked immunosorbent (ELISA) assay as previously described [25]. Briefly, 96‐well plates were coated with recombinant human IL13Rα2‐Fc (1 µg/mL; R&D Systems, Minneapolis, MN; Cat. 7147‐IR‐100) or IL13Rα1‐Fc (R&D Systems; Cat. 146‐IR‐100), 2% bovine serum albumin (BSA) in TBST, washed three times with TBST, and incubated with serial dilutions of hBTE. Bound proteins were detected using an anti‐6×His tag antibody (1:1000; Abcam, Cambridge, UK; Cat. AD1.1.10) followed by incubation with 1‐Step Slow TMB‐ELISA substrate (Thermo Fisher Scientific; Cat. 34022). The reaction was stopped with 2 N HCl, and absorbance was measured at 470 nm using a BioTek microplate reader (BioTek Instruments, Winooski, VT).

### 4.5 Flow Cytometry

Flow cytometry staining and analyses were as previously described [25]. Briefly, cells were resuspended in Dulbecco's phosphate‐buffered saline (DPBS; Corning Life Sciences, Corning, NY; Cat. 20‐031‐CV) supplemented with 2% fetal bovine serum (FBS; R&D Systems) and Human TruStain FcX reagent (1:75; BioLegend, San Diego, CA; Cat. 422302) to block nonspecific Fc receptor binding. Fluorochrome‐conjugated antibodies used for flow cytometry are listed in Table S3. For surface marker staining, cells were incubated with antibodies diluted in 2% FBS/DPBS for 30 min at 4°C. Dead cells were identified using Fixable Viability Dye eFluor 780 (1:1000 in DPBS; Invitrogen, Waltham, MA; Cat. 65‐0865‐18). For intracellular detection, cells were first labeled with the viability dye, then fixed and permeabilized using the eBioscience FOXP3/Transcription Factor Staining Buffer Set (Invitrogen; Cat. 00‐5523‐00) before incubation with intracellular antibodies. Samples were acquired on a BD FACSymphony A5 flow cytometer (Becton Dickinson, Franklin Lakes, NJ) at the Robert H. Lurie Comprehensive Cancer Center Flow Cytometry Core, and data were analyzed with FlowJo v10.9.0 (Becton Dickinson).

A gating strategy for all flow cytometric analysis is depicted in Figure S4.

### 4.6 Chromium‐51 Release Assay

Cytotoxicity of T cells against glioma cells in the presence of hBTE was assessed using a standard chromium‐51 (Cr51) release assay as previously described [25]. Briefly, T cells were isolated from human PBMCs with the EasySep Human T Cell Isolation Kit (STEMCELL Technologies, Vancouver, Canada; Cat. 100–0695). Target glioma cells were labeled with 0.05 mCi Cr‐51 (Revvity, Waltham, MA; Cat. NEZ030001MC) and subsequently co‐cultured with T cells at a 10:1 effector‐to‐target (E:T) ratio in the presence of graded hBTE concentrations. As a positive control, ImmunoCult Human CD3/CD28/CD2 T Cell Activator (STEMCELL Technologies; Cat. 10970) was included. After an 18‐h incubation, supernatants were collected into 96‐well LumaPlates (PerkinElmer, Waltham, MA; Cat. 6006633), dried, and counted for radioactivity using a gamma counter (PerkinElmer). Percent specific lysis was determined relative to spontaneous and maximum release (the latter obtained by lysing targets with 1% Triton X‐100). Data represent mean ± SEM from triplicate wells, and statistical significance was evaluated by Student's t‐test.

### 4.7 Magnetic Bead‐Based Multiplex Immunoassays

Cytokine concentrations (GM‐CSF, IFNγ, IL‐13, IL‐18, IL‐2, IL‐4, IL‐5, and TNFα) in cell culture supernatants were quantified using the ProcartaPlex Human Th1/Th2 Cytokine 11‐plex panel (Thermo Fisher Scientific, Waltham, MA; Cat. EPX1101081090) according to the manufacturer's instructions. Human T cells were isolated from peripheral blood mononuclear cells (PBMCs) obtained from two healthy donors (Stanford Blood Center) and two patients with GBM (Northwestern University Nervous System Tumor Bank) using the EasySep Human T Cell Isolation Kit (STEMCELL Technologies, Vancouver, Canada; Cat. 100–0695). Isolated T cells were co‐cultured with IL13RA2‐positive GBM6 or IL13RA2‐negative GBM39 cells at an effector‐to‐target (E:T) ratio of 10:1 in the presence of hBTE (200 ng/µL). After 48 h, cultures were centrifuged to pellet cells, and the resulting supernatants were filtered through a 0.2 µm syringe filter before cytokine analysis by the Northwestern University Comprehensive Metabolic Core.

### 4.8 Western Blot

The western blot analysis was performed as previously described with minor modifications [47]. Briefly, purified humanized antibodies were quantified using a Pierce BCA Protein Assay Kit (Thermo Fisher Scientific, Waltham, MA; Cat. A55864). For denaturing conditions, samples were mixed with NuPAGE LDS Sample Buffer (4X) (Invitrogen, Waltham, MA; Cat. NP0007) containing 2.5% 2‐mercaptoethanol (Sigma‐Aldrich, St. Louis, MO; Cat. M6250) and heated at 95°C for 10 min. For native conditions, samples were diluted in NativePAGE Sample Buffer (4X) (Invitrogen; Cat. BN2003). Proteins and molecular weight standards (Sigma‐Aldrich, Cat. S8445) were separated by SDS‐polyacrylamide gel electrophoresis (SDS‐PAGE) on Novex Tris‐Glycine Mini Gels (Thermo Fisher Scientific; Cat. XP04205BOX). Following electrophoresis, proteins were transferred onto a methanol‐activated 0.2 µm PVDF membrane (MilliporeSigma, Burlington, MA; Cat. IPVH08100) using the iBlot 3 Western Blot Transfer System (Thermo Fisher Scientific). Membranes were blocked in 5% nonfat dry milk prepared in Tris‐buffered saline with 0.1% Tween‐20 (TBST) (Boston BioProducts, Ashland, MA; Cat. IBB180) for 2 h at room temperature, then incubated for 2 h with goat anti‐human IgG (Fc‐specific) HRP‐conjugated secondary antibody (1:2000; Thermo Fisher Scientific; Cat. A18817). After TBST washes, blots were developed with Pierce ECL Western Blotting Substrate (Thermo Fisher Scientific; Cat. 32106) and imaged using the iBright CL750 Imaging System (Thermo Fisher Scientific).

### 4.9 Animal Studies

All animal procedures were approved by the Northwestern University Institutional Animal Care and Use Committee. Six‐ to 8‐week‐old male and female mice were group‐housed by sex and randomly assigned to treatment or control cohorts. PD GBM (GBM6, GBM12, GBM38, GBM39, GBM6r, GBM12r) and NCI‐H460 xenograft models were established in NSG‐MHC I/II DKO mice (Jackson Laboratory), whereas the MDA‐MB‐231Br3 brain metastasis model was performed in athymic nude mice (Jackson Laboratory) due to radiation intolerance in NSG‐MHC I/II DKO mice. All animals received perioperative hydration and analgesia according to institutional guidelines. Mice were engrafted with human PBMCs by intravenous injection, either before or after tumor implantation as described below. Specific hBTE treatment regimens and doses for each model are detailed in the corresponding figure legends and described in the Results section.

PD GBM tumors were established by stereotactic implantation of glioma cells (2.5 µL) into the right cerebral hemisphere as previously described [25]. 7 to 8 days after tumor implantation, mice received an intravenous injection of 7–10 × 10^6^ HLA‐matched human PBMCs. Treatments were administered four times per week for 3 weeks, and animals were monitored for survival. The experimental endpoint was defined as a 20% loss of initial body weight or onset of neurological symptoms. Brains from mice with stable disease were collected, and long‐term survivor (LTS) status was assigned to animals with no detectable tumor on histological evaluation.

A breast‐brain metastasis model was established in nude mice, in which 7.5 × 10^4^ MDA‐MB‐231Br3 cells were implanted via intracarotid injection using microsurgical techniques as previously described [78]. 7 to 8 days after tumor implantation, mice received an intravenous injection of 3 × 106 human T cells. To maintain T‐cell viability following PBMC engraftment, recombinant human interleukin‐2 (30 000 IU; Thermo Fisher) was administered intraperitoneally three times per week, beginning 24 h after PBMC injection and continuing throughout the study. Specific treatment regimens and doses are detailed in the corresponding figure legends and described in the Results section.

For lung subcutaneous xenograft studies, NSG‐MHC I/II DKO mice were engrafted with human PBMCs 1 week prior to tumor implantation. NCI‐H460 cells (3 × 10^5^) were suspended in a 1:1 mixture of PBS and Matrigel (Corning) and injected subcutaneously into the flank. Mice were inspected daily, and tumors were harvested on day 20 after implantation. Tumor weight and dimensions were measured and compared across groups.

### 4.10 Quantification of hBTE in a Breast Cancer Brain Metastatic Model

Cy5 labeling of hBTE was performed as previously described [27]. Metastases of MDA‐MB‐231Br cells expressing tdTomato were established via intracarotid infusion of cells into athymic nude mice as described in the animal studies section. Mice were divided into two groups: one received five cycles of fractionated irradiation (2 Gy per cycle, 10 Gy total), and the other served as a non‐irradiated control. After 48 h, both groups were administered a single intravenous dose of Cy5‐labeled hBTE (50 µg per animal). Blood vessels were visualized using fluorescein‐conjugated lectin (Vector Labs). At 24 h, mice were perfused, and brains were collected, frozen, sectioned into 1 mm slices, and imaged using a Nikon Eclipse AR1 confocal microscope (Nikon Co.) as described previously [27].

Images of metastases and surrounding tissue were analyzed in ImageJ (version 1.54f; Bitplane, Oxford Instruments). Each image contained three fluorescence channels: green for BiTE, red for tumor cells, and blue for blood vessels. A custom ImageJ macro automated image processing by splitting the channels, applying the default dark threshold to the red channel, and defining regions of interest (ROIs) that delineate tumor boundaries. The raw integrated density of the green channel signal located outside these ROIs was quantified to measure BiTE distribution relative to the tumor region.

### 4.11 Histology and Immunohistochemistry of Mouse and Human Samples

Details of all primary and secondary antibodies used in this study, including sources and dilutions, are provided in Table S3. Human glioblastoma samples were obtained from the Nervous System Tumor Bank at Northwestern Memorial Hospital under Institutional Review Board approval (IRB #STU0095863) and prepared as formalin‐fixed, paraffin‐embedded specimens for immunohistochemical analysis. Serial tissue sections were stained with hematoxylin and eosin (H&E) and subjected to immunohistochemistry staining for IL13RA2. The antigen retrieval (pH to 6) was performed per the manufacturer's protocol. Tissues were subsequently stained with goat anti‐human IL13RA2 antibody, followed by donkey anti‐goat HRP polymer. All samples were de‐identified prior to analysis. Immunohistochemical scoring was independently performed by a board‐certified neuropathologist (C.H.), who evaluated tumor cell staining according to the percentage of IL13RA2‐positive cells: 0, no staining; 1, ≤10%; 2, 10%–50%; and 3, ≥50%.

All formalin‐fixed brains and organs from intracranial PD GBM xenograft mouse models were processed by the Mouse Histology and Phenotyping Laboratory (MHPL) at the Robert H. Lurie Comprehensive Cancer Center of Northwestern University. Briefly, animals were perfused with PBS at defined endpoints or upon clinical decline, fixed in 10% neutral‐buffered formalin for 24 h at 4°C, paraffin‐embedded, sectioned (4 µm), and stained with H&E. For immunohistochemistry, serial sections were incubated with rabbit anti‐human CD3 and rabbit anti‐mouse GFAP, followed by anti‐rabbit HRP polymer. The brain and spinal cord sections were additionally stained with Luxol Fast Blue to assess myelin integrity. Histopathological evaluation of all organs for treatment‐related toxicity was independently performed by a board‐certified neuropathologist (C.H.).

Staining of the tissue sections from the formalin‐fixed brains of GBM38‐bearing mice was performed post endpoint to assess IL13RA2 expression. Briefly, animals were perfused with PBS at defined endpoints or upon clinical decline, fixed in 10% neutral‐buffered formalin for 24 h at 4°C, paraffin‐embedded, sectioned (4 µm), and stained for IL13RA2. Heat‐induced epitope retrieval (110°C, 10 min, pH 6) was performed per the manufacturer's protocol. Tissues were subsequently stained with rabbit anti‐human IL13RA2 antibody, followed by goat anti‐rabbit HRP polymer.

All formalin‐fixed NCI‐H460 subcutaneous xenograft models were processed by the Mouse Histology and Phenotyping Laboratory (MHPL) at the Robert H. Lurie Comprehensive Cancer Center of Northwestern University. Briefly, animals were sacrificed at defined endpoints, subcutaneous tumors were excised, fixed in 10% neutral‐buffered formalin for 24 h at 4°C, paraffin‐embedded, sectioned (4 µm), and stained with H&E. For immunohistochemistry, serial sections were incubated with anti‐human CD3 followed by anti‐rabbit HRP polymer. H&E‐ and CD3E‐stained tumor sections were digitized and analyzed in QuPath v0.5.1 to quantify viable and necrotic tissue areas as well as relative T cell abundance. Necrotic regions were identified using a pixel classifier trained on eight manually annotated necrotic and non‐necrotic areas within the tumor boundary. Classifier training was performed with QuPath's built‐in artificial neural network at moderate resolution using default multiscale feature settings. The percentage of necrosis was calculated based on the predicted necrotic pixel area within the defined tumor region of interest (ROI). Relative T cell abundance was quantified using QuPath's native positive cell detection plugin. CD3‐positive cells were defined using parameters defined in Table S4.

### 4.12 Automated Multiplexed Sequential Immunofluorescence Imaging

Automated multiplexed sequential immunofluorescence (seqIF) was performed as described previously [47]. Briefly, FFPE tumor sections were processed using the COMET system (Lunaphore Technologies). Primary antibodies included GFAP and IL13Rα2, with DAPI used for nuclear counterstaining (antibody details and dilutions listed in Table S3). All antibodies were pre‐validated by single‐plex immunostaining, and dilutions were optimized at the manufacturer's recommended concentration (MRD), MRD/2, and MRD/4 for optimal signal‐to‐noise. Alexa Fluor 555 and 647 secondary antibodies (ThermoFisher Scientific) were used at 1:200 and 1:400 dilutions, respectively. Multiplexed staining was performed using Lunaphore's automated seqIF protocol as previously described, involving iterative cycles of staining, imaging, and elution with no manual intervention. Primary and secondary antibody incubation, elution, quenching, and imaging steps followed the manufacturer's optimized conditions (Lunaphore Technologies). Following image acquisition, automated registration and alignment were performed using COMET Control Software, and multiplexed images were visualized and pseudo‐colored in HORIZON Viewer (Lunaphore Technologies).

### 4.13 Analysis of Publicly Available RNA Sequencing Datasets

Levels of IL13RA2 gene expression in normal adult tissues were analyzed using bulk RNA sequencing data from the adult GTEx RNA sequencing data (V10) [28], available at https://www.gtexportal.org/home/downloads/adult‐gtex/bulk_tissue_expression. IL13RA2 expression in glioma was analyzed using bulk RNA sequencing data from the TCGA‐GBM and TCGA‐LGG datasets, which were downloaded from the TCGA Data Portal at https://www.cancer.gov/ccg/research/genome‐sequencing/tcga. Analysis of IL13RA2 expression within the GBM tumor microenvironment was performed using the harmonized single‐cell GBM atlas (GBmap), which includes 338 564 cells from 110 patient samples and is accessible through CellxGene at https://cellxgene.cziscience.com/collections/999f2a15‐3d7e‐440b‐96ae‐2c806799c08c. A microarray dataset containing 23 matched primary and recurrent GBM samples, GSE4271 [79], was obtained from the Gene Expression Omnibus (GEO) database at https://www.ncbi.nlm.nih.gov/geo/query/acc.cgi?acc = GSE4271.

### 4.14 Statistical Analyses

All statistical analyses were performed in R (v4.5.1) using the packages listed in Table S4. Cr51 differences in normalized viability were evaluated by two‐way ANOVA (factors: Group and Concentration); the reported p‐value corresponds to the main effect of group. Differences in lung cancer histology and veterinary clinical chemistry parameters were evaluated using an unpaired Welch's *t*‐test (unequal variances assumed). Survival curves were generated using the Kaplan–Meier method and compared by log‐rank test relative to saline controls.

## Author Contributions

IVB conceived and funded the study. JTD and IVB designed experiments. JTD, AMR, BEH, AT, MFC, SM, MZ, performed the experiments. VPK provided human lung fibroblasts, and KM assisted with the procurement of patients’ tissue sections and PBMC. JTD and JRP analyzed the data. JTD, BEH, ARM, and IVB wrote the manuscript. JTD, and IVB revised the manuscript. CMH conducted immunohistochemical scoring of IL13RA2 expression and histopathological evaluation of organs. DS performed confocal microscopy of tissues. CDJ provided a PD glioma xenograft model and reviewed the manuscript.ML and RS advised and reviewed the manuscript. All authors met authorship criteria and approved the final manuscript. IVB is the guarantor of the work.

## Conflicts of Interest

Balyasnikova, I.V. is an inventor of humanized IL13RA2 antibody^33^. All other authors declare no conflict of interest.

## Supporting information




**Supporting File**: advs75477‐sup‐0001‐SuppMat.docx.

**Supporting Table**: advs75477‐sup‐0002‐TableS1‐S5.xlsx.

## Data Availability

The data that support the findings of this study are available from the corresponding author upon reasonable request.

## References

[1] A. Viardot , M. E. Goebeler , G. Hess , et al., “Phase 2 Study of the Bispecific T‐cell Engager (BiTE) Antibody Blinatumomab in Relapsed/Refractory Diffuse Large B‐Cell Lymphoma,” Blood 127, no. 11 (2016): 1410–1416, 10.1182/blood-2015-06-651380. 26755709 PMC4797019

[2] A. S. Advani , A. Moseley , K. M. O'Dwyer , et al., “SWOG 1318: A Phase II Trial of Blinatumomab Followed by POMP Maintenance in Older Patients With Newly Diagnosed Philadelphia Chromosome–Negative B‐Cell Acute Lymphoblastic Leukemia,” Journal of Clinical Oncology 40, no. 14 (2022): 1574–1582, 10.1200/jco.21.01766. 35157496 PMC9084435

[3] A. Y. Krishnan , S. Manier , E. Terpos , et al., “MajesTEC‐7: A Phase 3, Randomized Study of Teclistamab + Daratumumab + Lenalidomide (Tec‐DR) Versus Daratumumab + Lenalidomide + Dexamethasone (DRd) in Patients With Newly Diagnosed Multiple Myeloma Who Are either Ineligible or Not Intended for Autologous Stem Cell Transplant,” Blood 140, no. 1 (2022): 10148–10149, 10.1182/blood-2022-160173.

[4] P. Moreau , A. L. Garfall , N. van de Donk , et al., “Teclistamab in Relapsed or Refractory Multiple Myeloma,” New England Journal of Medicine 387, no. 6 (2022): 495–505, 10.1056/NEJMoa2203478. 35661166 PMC10587778

[5] J. C. Hassel , S. Piperno‐Neumann , P. Rutkowski , et al., “Three‐Year Overall Survival With Tebentafusp in Metastatic Uveal Melanoma,” New England Journal of Medicine 389, no. 24 (2023): 2256–2266, 10.1056/NEJMoa2304753. 37870955 PMC11188986

[6] R. Stupp , W. P. Mason , M. J. van den Bent , et al., “Radiotherapy Plus Concomitant and Adjuvant Temozolomide for Glioblastoma,” New England Journal of Medicine 352, no. 10 (2005): 987–996, 10.1056/NEJMoa043330. 15758009

[7] R. Stupp , S. Taillibert , A. Kanner , et al., “Effect of Tumor‐Treating Fields Plus Maintenance Temozolomide vs Maintenance Temozolomide Alone on Survival in Patients With Glioblastoma,” Jama 318, no. 23 (2017): 2306–2316, 10.1001/jama.2017.18718. 29260225 PMC5820703

[8] L. R. Schaff and I. K. Mellinghoff , “Glioblastoma and Other Primary Brain Malignancies in Adults,” Jama 329, no. 7 (2023): 574–587, 10.1001/jama.2023.0023. 36809318 PMC11445779

[9] C. M. Walton , M. Bell , R. O'Neil , et al., “Chimeric Antigen Receptor (CAR) T‐Cell Therapy for Glioblastoma (GBM): Current Clinical Insights, Challenges, and Future Directions,” Journal for ImmunoTherapy of Cancer 13, no. 10 (2025): 012308, 10.1136/jitc-2025-012308. PMC12581071 41176315

[10] S. Al Hadidi , H. E. Heslop , M. K. Brenner , and M. Suzuki , “Bispecific Antibodies and Autologous Chimeric Antigen Receptor T Cell Therapies for Treatment of Hematological Malignancies,” Molecular Therapy 32, no. 8 (2024): 2444–2460, 10.1016/j.ymthe.2024.05.039. 38822527 PMC11405165

[11] P. C. Gedeon , B. D. Choi , T. R. Hodges , M. DA , D. D. Bigner , and J. H. Sampson , “An EGFRvIII‐targeted Bispecific T‐cell Engager Overcomes Limitations of the Standard of Care for Glioblastoma,” Expert Review of Clinical Pharmacology 6, no. 4 (2013): 375–386, 10.1586/17512433.2013.811806. 23927666 PMC4034273

[12] P. C. Gedeon , T. H. Schaller , S. K. Chitneni , et al., “A Rationally Designed Fully Human EGFRvIII:CD3‐Targeted Bispecific Antibody Redirects Human T Cells to Treat Patient‐Derived Intracerebral Malignant Glioma,” Clinical Cancer Research 24, no. 15 (2018): 3611–3631, 10.1158/1078-0432.Ccr-17-0126. 29703821 PMC6103776

[13] R. Sun , Y. Zhou , L. Han , et al., “A Rational Designed Novel Bispecific Antibody for the Treatment of GBM,” Biomedicines 9, no. 6 (2021): 640, 10.3390/biomedicines9060640. 34204931 PMC8230177

[14] M. E. Goebeler and R. C. Bargou , “T Cell‐Engaging Therapies — BiTEs and Beyond,” Nature Reviews Clinical Oncology 17, no. 7 (2020): 418–434, 10.1038/s41571-020-0347-5. 32242094

[15] D. Zagzag , K. Salnikow , L. Chiriboga , et al., “Downregulation of Major Histocompatibility Complex Antigens in Invading Glioma Cells: Stealth Invasion of the Brain,” Laboratory Investigation 85, no. 3 (2005): 328–341, 10.1038/labinvest.3700233. 15716863

[16] K. Dhatchinamoorthy , J. D. Colbert , and K. L. Rock , “Cancer Immune Evasion Through Loss of MHC Class I Antigen Presentation,” Frontiers in Immunology 12 (2021): 636568, 10.3389/fimmu.2021.636568. 33767702 PMC7986854

[17] C. T. Harris and S. Cohen , “Reducing Immunogenicity by Design: Approaches to Minimize Immunogenicity of Monoclonal Antibodies,” Biodrugs 38, no. 2 (2024): 205–226, 10.1007/s40259-023-00641-2. 38261155 PMC10912315

[18] Z. Hu , S. Cohen , and S. J. Swanson , “The Immunogenicity of Human‐Origin Therapeutic Antibodies Are Associated With V Gene Usage,” Frontiers in Immunology 14 (2023): 2023, 10.3389/fimmu.2023.1237754. PMC10502710 37720227

[19] W. Debinski , N. I. Obiri , S. K. Powers , I. Pastan , and R. K. Puri , “Human Glioma Cells Overexpress Receptors for Interleukin 13 and Are Extremely Sensitive to a Novel Chimeric Protein Composed of Interleukin 13 and Pseudomonas Exotoxin,” Clinical Cancer Research 1, no. 11 (1995): 1253–1258. 9815919

[20] J. S. Jarboe , K. R. Johnson , Y. Choi , R. R. Lonser , and J. K. Park , “Expression of Interleukin‐13 Receptor α2 in Glioblastoma Multiforme: Implications for Targeted Therapies,” Cancer Research 67, no. 17 (2007): 7983–7986, 10.1158/0008-5472.Can-07-1493. 17804706

[21] B. H. Joshi , G. E. Plautz , and R. K. Puri , “Interleukin‐13 Receptor Alpha Chain: A Novel Tumor‐Associated Transmembrane Protein in Primary Explants of Human Malignant Gliomas,” Cancer Research 60, no. 5 (2000): 1168–1172. 10728667

[22] W. Debinski , B. Slagle , D. M. Gibo , S. K. Powers , and G. Y. Gillespie , “Expression of a Restrictive Receptor for Interleukin 13 Is Associated with Glial Transformation,” Journal of Neuro‐Oncology 48, no. 2 (2000): 103–111, 10.1023/a:1006446426611. 11083073

[23] C. E. Brown , C. D. Warden , R. Starr , et al., “Glioma IL13Rα2 Is Associated with Mesenchymal Signature Gene Expression and Poor Patient Prognosis,” PLoS ONE 8, no. 10 (2013): 77769, 10.1371/journal.pone.0077769. PMC3800130 24204956

[24] C. E. Brown , J. C. Hibbard , D. Alizadeh , et al., “Locoregional Delivery of IL‐13Rα2‐Targeting CAR‐T Cells in Recurrent High‐grade Glioma: A Phase 1 Trial,” Nature Medicine 30 (2024): 1001–1012, 10.1038/s41591-024-02875-1. PMC11031404 38454126

[25] K. C. Pituch , M. Zannikou , L. Ilut , et al., “Neural Stem Cells Secreting Bispecific T Cell Engager to Induce Selective Antiglioma Activity,” Proceedings of the National Academy of Sciences 118, no. 9 (2021): 2015800118, 10.1073/pnas.2015800118. PMC7936285 33627401

[26] K. C. Pituch , J. Miska , G. Krenciute , et al., “Adoptive Transfer of IL13Rα2‐Specific Chimeric Antigen Receptor T Cells Creates a Pro‐inflammatory Environment in Glioblastoma,” Molecular Therapy 26, no. 4 (2018): 986–995, 10.1016/j.ymthe.2018.02.001. 29503195 PMC6079480

[27] Y. Feng , B. Haupt , T. T. Huynh , et al., “Longitudinal Imaging Reveals Tumor Uptake and Prolonged Retention of Bispecific T Cell–Engaging Antibody in GBM via Passive and Active Mechanisms,” Clinical Cancer Research 31, no. 16 (2025): 3537–3549, 10.1158/1078-0432.CCR-24-4194. 40512178 PMC12228081

[28] J. Lonsdale , J. Thomas , M. Salvatore , et al., “The Genotype‐Tissue Expression (GTEx) Project,” Nature Genetics 45, no. 6 (2013): 580–585, 10.1038/ng.2653. 23715323 PMC4010069

[29] N. Pedano , A. E. Flanders , L. Scarpace , et al., “The Cancer Genome Atlas Low Grade Glioma Collection (TCGA‐LGG) (Version 3) [Data set],” The Cancer Imaging Archive (2016), 10.7937/K9/TCIA.2016.L4LTD3TK.

[30] L. Scarpace , T. Mikkelsen , S. Cha , et al., “The Cancer Genome Atlas Glioblastoma Multiforme Collection (TCGA‐GBM) (Version 5) [Data set],” The Cancer Imaging Archive (2016), 10.7937/K9/TCIA.2016.RNYFUYE9.

[31] C. Ruiz‐Moreno , S. M. Salas , E. Samuelsson , et al., “Charting the Single‐Cell and Spatial Landscape of IDH‐Wild‐Type Glioblastoma With GBmap,” Neuro‐Oncology 27 (2025): 2281–2295, 10.1093/neuonc/noaf113. 40312969 PMC12526130

[32] I. V. Balyasnikova , D. A. Wainwright , E. Solomaha , et al., “Characterization and Immunotherapeutic Implications for a Novel Antibody Targeting Interleukin (IL)‐13 Receptor α2,” Journal of Biological Chemistry 287, no. 36 (2012): 30215–30227, 10.1074/jbc.M112.370015. 22778273 PMC3436275

[33] I. V. Balyasnikova , “Humanized Antibody Targeting the Tumor‐Associated Antigen IL13RA2,” US patent US20230312731A1. 2023.

[34] T. Chitnis , B. J. Kaskow , J. Case , et al., “Nasal Administration of Anti‐CD3 Monoclonal Antibody Modulates Effector CD8+ T Cell Function and Induces a Regulatory Response in T Cells in human Subjects,” Frontiers in Immunology 13 (2022): 956907, 10.3389/fimmu.2022.956907. 36505477 PMC9727230

[35] C. J. van der Woude , P. Stokkers , A. A. van Bodegraven , et al., “Phase I, Double‐Blind, Randomized, Placebo‐Controlled, Dose‐Escalation Study of NI‐0401 (a Fully Human Anti‐CD3 Monoclonal Antibody) in Patients With Moderate to Severe Active Crohnʼs Disease†,” Inflammatory Bowel Diseases 16, no. 10 (2010): 1708–1716, 10.1002/ibd.21252. 20848453

[36] C. Chang , V. S. Chavarro , J. V. E. Gerstl , et al., “Recurrent Glioblastoma—Molecular Underpinnings and Evolving Treatment Paradigms,” International Journal of Molecular Sciences 25, no. 12 (2024): 6733, 10.3390/ijms25126733. 38928445 PMC11203521

[37] P. Daniel , B. Meehan , S. Sabri , et al., “Detection of Temozolomide‐induced Hypermutation and Response to PD‐1 Checkpoint Inhibitor in Recurrent Glioblastoma,” Neuro‐Oncology Advances 4, no. 1 (2022), 10.1093/noajnl/vdac076. PMC9252128 35795471

[38] M. Meleiro and R. Henrique , “Epigenetic Alterations in Glioblastoma Multiforme as Novel Therapeutic Targets: A Scoping Review,” International Journal of Molecular Sciences 26, no. 12 (2025): 5634, 10.3390/ijms26125634. 40565099 PMC12192589

[39] Q. Wang , B. Hu , X. Hu , et al., “Tumor Evolution of Glioma‐Intrinsic Gene Expression Subtypes Associates with Immunological Changes in the Microenvironment,” Cancer Cell 32, no. 1 (2017): 42–56.e6, 10.1016/j.ccell.2017.06.003. 28697342 PMC5599156

[40] S. Yip , J. Miao , D. P. Cahill , et al., “MSH6 mutations Arise in Glioblastomas during Temozolomide Therapy and Mediate Temozolomide Resistance,” Clinical Cancer Research 15, no. 14 (2009): 4622–4629, 10.1158/1078-0432.Ccr-08-3012. 19584161 PMC2737355

[41] M. McCord , E. Bartom , K. Burdett , et al., “Modeling Therapy‐Driven Evolution of Glioblastoma with Patient‐Derived Xenografts,” Cancers 14, no. 22 (2022): 5494, 10.3390/cancers14225494. 36428586 PMC9688760

[42] M. Jaén , Á. Martín‐Regalado , R. A. Bartolomé , J. Robles , and J. I. Casal , “Interleukin 13 Receptor Alpha 2 (IL13Rα2): Expression, Signaling Pathways and Therapeutic Applications in Cancer,” Biochimica et Biophysica Acta (BBA) ‐ Reviews on Cancer 1877, no. 5 (2022): 188802, 10.1016/j.bbcan.2022.188802. 36152905

[43] E. Hart , Z. Odé , M. P. P. Derieppe , et al., “Blood‐brain Barrier Permeability Following Conventional Photon Radiotherapy – A Systematic Review and Meta‐analysis of Clinical and Preclinical Studies,” Clinical and Translational Radiation Oncology 35 (2022): 44–55, 10.1016/j.ctro.2022.04.013. 35601799 PMC9117815

[44] F. Teng , C. I. Tsien , T. S. Lawrence , and Y. Cao , “Blood–Tumor Barrier Opening Changes in Brain Metastases From Pre to One‐Month Post Radiation Therapy,” Radiotherapy and Oncology 125, no. 1 (2017): 89–93, 10.1016/j.radonc.2017.08.006. 28835339

[45] S. Cha , J. M. Lupo , M. H. Chen , et al., “Differentiation of Glioblastoma Multiforme and Single Brain Metastasis by Peak Height and Percentage of Signal Intensity Recovery Derived from Dynamic Susceptibility‐weighted Contrast‐enhanced Perfusion MR Imaging,” American Journal of Neuroradiology 28, no. 6 (2007): 1078–1084, 10.3174/ajnr.A0484. 17569962 PMC8134129

[46] S. J. Bagley , M. Logun , J. A. Fraietta , et al., “Intrathecal Bivalent CAR T Cells Targeting EGFR and IL13Rα2 in Recurrent Glioblastoma: Phase 1 Trial Interim Results,” Nature Medicine 30, no. 5 (2024): 1320–1329, 10.1038/s41591-024-02893-z. PMC13123313 38480922

[47] M. Zannikou , J. T. Duffy , D. Procissi , et al., “Bi‐specific T Cell‐Engaging Antibody Triggers Protective Immune Memory and Glioma Microenvironment Remodeling in Immune‐Competent Preclinical Models,” Journal for ImmunoTherapy of Cancer 13, no. 10 (2025): 011714, 10.1136/jitc-2025-011714. PMC12570949 41151835

[48] J. A. Kimball , D. J. Norman , C. F. Shield , et al., “The OKT3 Antibody Response Study: A Multicentre Study of Human Anti‐Mouse Antibody (HAMA) Production Following OKT3 Use in Solid Organ Transplantation,” Transplant Immunology 3, no. 3 (1995): 212–221, 10.1016/0966-3274(95)80027-1. 8581409

[49] M. B. Khazaeli , R. M. Conry , and A. F. LoBuglio , “Human Immune Respone to Monoclonal Antibodies,” Journal of Immunotherapy 15, no. 1 (1994): 42–52. 8110730 10.1097/00002371-199401000-00006

[50] R. Asano , K. Nagai , K. Makabe , et al., “Structural Considerations for Functional Anti‐EGFR × Anti‐CD3 Bispecific Diabodies in Light of Domain Order and Binding Affinity,” Oncotarget 9, no. 17 (2018): 13884–13893, 10.18632/oncotarget.24490. 29568402 PMC5862623

[51] C. Carrasco‐Padilla , A. Hernaiz‐Esteban , L. Álvarez‐Vallina , O. Aguilar‐Sopeña , and P. Roda‐Navarro , “Bispecific Antibody Format and the Organization of Immunological Synapses in T Cell‐Redirecting Strategies for Cancer Immunotherapy,” Pharmaceutics 15, no. 1 (2022): 132, 10.3390/pharmaceutics15010132. 36678761 PMC9863865

[52] A. Elsayed , L. Plüss , L. Nideroest , et al., “Optimizing the Design and Geometry of T Cell–Engaging Bispecific Antibodies Targeting CEA in Colorectal Cancer,” Molecular Cancer Therapeutics 23, no. 7 (2024): 1010–1020, 10.1158/1535-7163.Mct-23-0766. 38638035

[53] H. P. Loh , F. B. Mahfut , S. W. Chen , et al., “Manufacturability and Functionality Assessment of Different Formats of T‐cell Engaging Bispecific Antibodies,” MAbs 15, no. 1 (2023): 2231129, 10.1080/19420862.2023.2231129. 37403264 PMC10324450

[54] L. P. E. Z. J. Arribas , A. M. ROMÁN , I. E. A. GRINYÓ , S. DURO SÁNCHEZ , R. I. RIUS , and T. V. NOGALES , “Immune Cells Expressing Chimeric Antigen Receptors and Bispecific Antibodies and Uses Thereof,” patent WO2023186873. 2023 Oct 5.

[55] A. Löffler , P. Kufer , R. Lutterbüse , et al., “A Recombinant Bispecific Single‐chain Antibody, CD19 × CD3, Induces Rapid and High Lymphoma‐directed Cytotoxicity by Unstimulated T Lymphocytes,” Blood 95, no. 6 (2000): 2098–2103, 10.1182/blood.V95.6.2098. 10706880

[56] P. R. Lockman , R. K. Mittapalli , K. S. Taskar , et al., “Heterogeneous Blood–Tumor Barrier Permeability Determines Drug Efficacy in Experimental Brain Metastases of Breast Cancer,” Clinical Cancer Research 16, no. 23 (2010): 5664–5678, 10.1158/1078-0432.Ccr-10-1564. 20829328 PMC2999649

[57] D. H. Murrell , A. M. Hamilton , C. L. Mallett , R. van Gorkum , A. F. Chambers , and P. J. Foster , “Understanding Heterogeneity and Permeability of Brain Metastases in Murine Models of HER2‐Positive Breast Cancer through Magnetic Resonance Imaging: Implications for Detection and Therapy,” Translational Oncology 8, no. 3 (2015): 176–184, 10.1016/j.tranon.2015.03.009. 26055175 PMC4487267

[58] C. D. Arvanitis , V. Askoxylakis , Y. Guo , et al., “Mechanisms of Enhanced Drug Delivery in Brain Metastases With Focused Ultrasound‐Induced Blood–Tumor Barrier Disruption,” Proceedings of the National Academy of Sciences 115, no. 37 (2018): E8717–e8726, 10.1073/pnas.1807105115. PMC6140479 30150398

[59] M. Osswald , J. Blaes , Y. Liao , et al., “Impact of Blood–Brain Barrier Integrity on Tumor Growth and Therapy Response in Brain Metastases,” Clinical Cancer Research 22, no. 24 (2016): 6078–6087, 10.1158/1078-0432.Ccr-16-1327. 27521448

[60] D. Procissi , S. A. Jannetti , M. Zannikou , et al., “Low‐Level Whole‐Brain Radiation Enhances Theranostic Potential of Single‐Domain Antibody Fragments for Human Epidermal Growth Factor Receptor Type 2 (HER2)‐Positive Brain Metastases,” Neuro‐Oncology Advances 4, no. 1 (2022): vdac135, 10.1093/noajnl/vdac135. 36128586 PMC9476215

[61] S. Zhao , Y. Chen , P. S. Bhojnagarwala , et al., “Targeting IL13Rα2 in Melanoma with a Bispecific T‐cell Engager: Expression Profiling and Preclinical Evaluation,” Journal for ImmunoTherapy of Cancer 13, no. 6 (2025): 011073, 10.1136/jitc-2024-011073. PMC12161313 40484645

[62] A. Ceschi , R. Noseda , K. Palin , and K. Verhamme , “Immune Checkpoint Inhibitor‐Related Cytokine Release Syndrome: Analysis of WHO Global Pharmacovigilance Database,” Frontiers in Pharmacology 11 (2020): 557, 10.3389/fphar.2020.00557. 32425791 PMC7212758

[63] S. J. Rotz , D. Leino , S. Szabo , J. L. Mangino , B. K. Turpin , and J. G. Pressey , “Severe Cytokine Release Syndrome in a Patient Receiving PD‐1‐Directed Therapy,” Pediatric Blood & Cancer 64, no. 12 (2017): 26642 , 10.1002/pbc.26642. 28544595

[64] M. Subklewe , “BiTEs Better Than CAR T Cells,” Blood Advances 5, no. 2 (2021): 607–612, 10.1182/bloodadvances.2020001792. 33496755 PMC7839370

[65] S. H. Tay , M. M. X. Toh , Y. L. Thian , et al., “Cytokine Release Syndrome in Cancer Patients Receiving Immune Checkpoint Inhibitors: A Case Series of 25 Patients and Review of the Literature,” Frontiers in Immunology 13 (2022): 807050, 10.3389/fimmu.2022.807050. 35154124 PMC8831742

[66] M. Yomota , K. Mirokuji , M. Sakaguchi , et al., “Cytokine Release Syndrome Induced by Immune‐Checkpoint Inhibitor Therapy for Non‐Small‐Cell Lung Cancer,” Internal Medicine 60, no. 21 (2021): 3459–3462, 10.2169/internalmedicine.5922-20. 33775995 PMC8627810

[67] M. Golmohammadi , S. Raza , M. Albayyadhi , et al., “Comprehensive Assessment of Adverse Event Profiles Associated With Bispecific Antibodies in Multiple Myeloma,” Blood Cancer Journal 15, no. 1 (2025): 130, 10.1038/s41408-025-01334-5. 40750760 PMC12316967

[68] J. Lantz , N. Pham , C. Jones , D. Reed , F. El Chaer , and M. Keng , “Blinatumomab in Practice,” Current Hematologic Malignancy Reports 19, no. 1 (2024): 1–8, 10.1007/s11899-023-00714-7. 38060085

[69] R. Iurlaro , I. Waldhauer , E. Planas‐Rigol , et al., “A Novel EGFRvIII T‐Cell Bispecific Antibody for the Treatment of Glioblastoma,” Molecular Cancer Therapeutics 21, no. 10 (2022): 1499–1509, 10.1158/1535-7163.Mct-22-0201. 35915983

[70] Y. Feng , K. Xie , Y. Yin , et al., “A Novel Anti‐B7‐H3 × Anti‐CD3 Bispecific Antibody with Potent Antitumor Activity,” Life 12, no. 2 (2022): 157, 10.3390/life12020157. 35207448 PMC8879513

[71] R. A. Vaubel , S. Tian , D. Remonde , et al., “Genomic and Phenotypic Characterization of a Broad Panel of Patient‐Derived Xenografts Reflects the Diversity of Glioblastoma,” Clinical Cancer Research 26, no. 5 (2020): 1094–1104, 10.1158/1078-0432.Ccr-19-0909. 31852831 PMC7056576

[72] R. Cailleau , R. Young , M. Olivé , and W. J. Reeves , “Breast Tumor Cell Lines From Pleural Effusions2,” JNCI: Journal of the National Cancer Institute 53, no. 3 (1974): 661–674, 10.1093/jnci/53.3.661. 4412247 PMC7364228

[73] T. Yoneda , P. J. Williams , T. Hiraga , M. Niewolna , and R. Nishimura , “A Bone‐Seeking Clone Exhibits Different Biological Properties From the MDA‐MB‐231 Parental human Breast Cancer Cells and a Brain‐Seeking Clone In Vivo and In Vitro,” Journal of Bone and Mineral Research 16, no. 8 (2001): 1486–1495, 10.1359/jbmr.2001.16.8.1486. 11499871

[74] M. Brower , D. N. Carney , H. K. Oie , A. F. Gazdar , and J. D. Minna , “Growth of Cell Lines and Clinical Specimens of Human Non‐Small Cell Lung Cancer in a Serum‐Free Defined Medium,” Cancer Research 46, no. 2 (1986): 798–806. 3940644

[75] DCTD TUMOR REPOSITORY , A Catalog of In Vitro Cell Lines, Transplantable Animal and Human Tumors, Canine Specimens and Yeast (Division of Cancer Treatment and Diagnosis, National Cancer Institute, 2013).

[76] J. F. Evans , O. A. Ledwell , Y. Tang , et al., “The Bi‐Steric Inhibitor RMC‐5552 Reduces mTORC1 Signaling and Growth in Lymphangioleiomyomatosis,” American Journal of Respiratory Cell and Molecular Biology 72, no. 6 (2025): 643–652, 10.1165/rcmb.2024-0242OC. 39531634 PMC12143409

[77] H. Einsele , H. Borghaei , R. Z. Orlowski , et al., “The BiTE (Bispecific T‐Cell Engager) Platform: Development and Future Potential of a Targeted Immuno‐Oncology Therapy Across Tumor Types,” Cancer 126, no. 14 (2020): 3192–3201, 10.1002/cncr.32909. 32401342

[78] A. Cordero , M. D. Ramsey , D. Kanojia , et al., “Combination of Tucatinib and Neural Stem Cells Secreting Anti‐HER2 Antibody Prolongs Survival of Mice with Metastatic Brain Cancer,” Proceedings of the National Academy of Sciences 119, no. 1 (2022) , 10.1073/pnas.2112491119. PMC8740706 34969858

[79] H. S. Phillips , S. Kharbanda , R. Chen , et al., “Molecular Subclasses of High‐Grade Glioma Predict Prognosis, Delineate a Pattern of Disease Progression, and Resemble Stages in Neurogenesis,” Cancer Cell 9, no. 3 (2006): 157–173, 10.1016/j.ccr.2006.02.019. 16530701

