# Supplementary material for: Fully Humanized Bispecific T Cell Engager Shows Potent Activity in Central Nervous System and Peripheral Tumors
Source: Adv Sci (Weinh). 2026 Apr 30;13(35):e22391. doi: 10.1002/advs.202522391 (PMC13292153; doi:10.1002/advs.202522391)
Supplement: Supplementary file 1 — Supporting File: advs75477‐sup‐0001‐SuppMat.docx. [file ADVS-13-e22391-s002.docx]

**Fully Humanized Bispecific T Cell Engager Shows Potent Activity in Central Nervous System and Peripheral Tumors.**

Joseph T. Duffy^1^, Angela Martin-Regalado^1^, Benedikt E. Haupt^1^, Jacob R. Pogue^1^, Aditi Thakur^1^, Manuel Fierro Cota^1^, Markella Zannikou^1^, Sol Misener^3^, Vera P. Krymskaya^4^, Kathleen McCortney ^1^, Jason Miska ^1^, Craig Horbinski^5^, Dmitri Simberg ^6,7^, Maciej S. Lesniak^1^, Charles D. James^1^, Roger Stupp ^1^, Irina V. Balyasnikova^1^*

^1^Department of Neurological Surgery, Northwestern University, Chicago, IL 60611, USA; Northwestern Medicine Malnati Brain Tumor Institute of the Lurie Comprehensive Cancer Center, Feinberg School of Medicine, Northwestern University, Chicago, IL 60611, USA.

^2^Department of Pathology, Northwestern University, Chicago, IL 60611, USA; Northwestern Medicine Malnati Brain Tumor Institute of the Lurie Comprehensive Cancer Center, Feinberg School of Medicine, Northwestern University, Chicago, IL 60611, USA.

^3^Feinberg Cardiovascular and Renal Research Institute, Northwestern University, Chicago, IL 60611, USA

^4^Department of Medicine, Perelman School of Medicine, University of Pennsylvania, Philadelphia, PA 19104, USA

^5^Department of Laboratory Medicine & Pathology, Mayo Clinic Florida, Jacksonville, FL 32224, USA

^6^Department of Pharmaceutical Sciences, Skaggs School of Pharmacy and Pharmaceutical Sciences, Aurora, CO 80045, USA

^7^Colorado Center for Nanomedicine and Nanosafety, University of Colorado Anschutz Medical Campus, Aurora, CO 80045, USA

 *Correspondence:

Irina V. Balyasnikova, Ph.D.

Department of Neurological Surgery,

Northwestern University,

Chicago, IL, 60611, USA

[irinabal@northwestern.edu](mailto:irinabal@northwestern.edu)

**
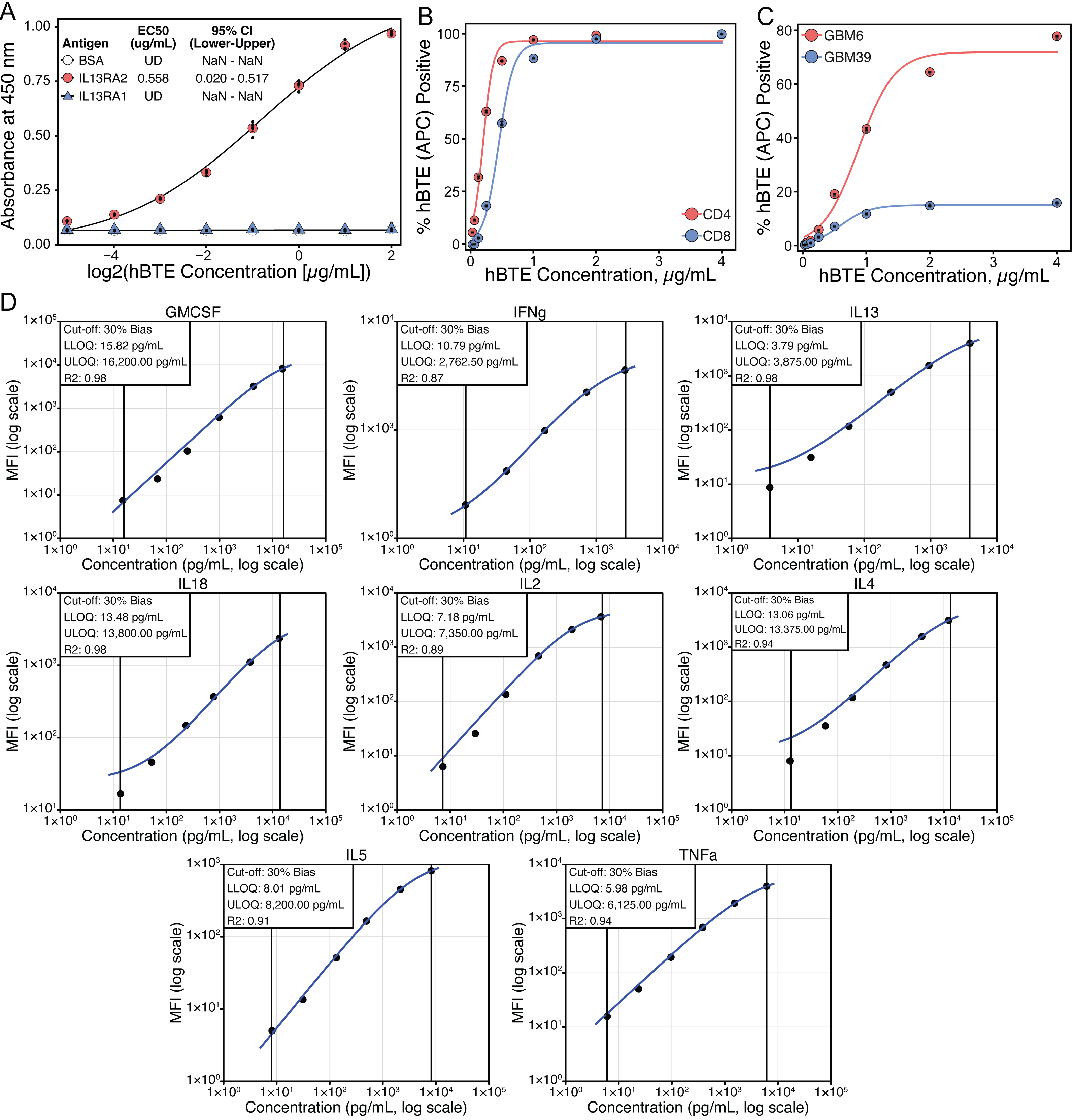
Supplemental Figure 1.** (A) ELISA measuring binding of hBTE to human IL13RA2, human IL13RA1, or the BSA control. EC50 values indicate IL13RA2-specific binding. UD, undetectable. (B) Flow cytometry percent-positive cells showing dose-dependent hBTE binding to CD4 and CD8 T cells using an AF647-conjugated anti-His-tag antibody. (C) Flow cytometry showing dose-dependent hBTE binding to IL13RA2-positive GBM6 cells, but not to IL13RA2-negative GBM39 cells, using an AF647-conjugated anti-His tag antibody. (D) Standard curves validating accurate detection of eight cytokines included in the multiplex assay: IL-2, IL-4, IL-5, IL-13, IL-18, IFN-γ, TNF-α, and GM-CSF. LLOQ, lower limit of quantification. ULOQ, upper limit of quantification.

**
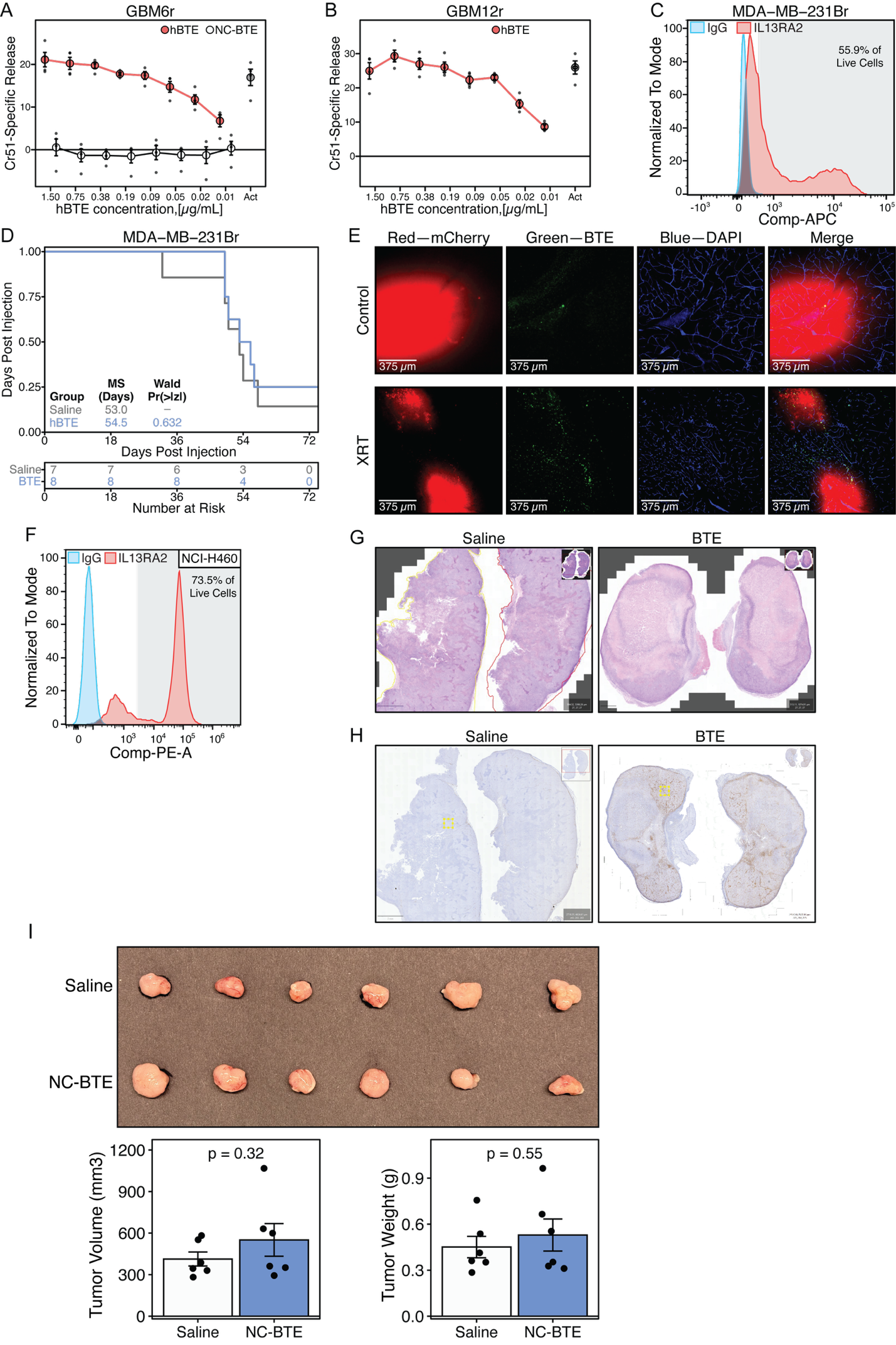
Supplemental Figure 2.** (A–B) Chromium-51 release assays showing dose-dependent T cell–mediated killing of (A) GBM6r and (B) GBM12r cells following treatment with hBTE (n = 4) or NC-BTE (n = 4). Statistical significance between treatments was determined by two-way ANOVA. (C) Flow cytometry analysis of IL13RA2 surface expression on MDA-MB-231br, a triple-negative breast cancer brain metastatic cell line (55.9% IL13RA2+), detected using APC-conjugated anti-human IL13RA2 antibody. (D) Survival curves of nude mice bearing intracranial MDA-MB-231Br tumors treated with hBTE (50 µg, i.v., twice weekly) or saline. MS, median survival. Statistical significance was determined by the log-rank test relative to the saline control. (E) Immunofluorescent single-channel images of brain sections from control and XRT-treated mice following hBTE administration. red, mCherry (tumor); green, hBTE; blue, DAPI. Scale bar, 375 µm. (F) Flow cytometry analysis of IL13RA2 surface expression on NCI-H460, an extracranial lung cancer cell line (73.5% IL13RA2+), detected using PE-conjugated anti-human IL13RA2 antibody. (G–H) Histological analysis of NCI-H460 flank tumors from NSG MHC I/II DKO mice treated with hBTE. (G) Hematoxylin and eosin (H&E) staining and (H) CD3 immunostaining. (I) Tumor growth dynamics NCI-H460 flank tumors from NSG MHC I/II DKO mice treated with Saline and NC-BTE.

**
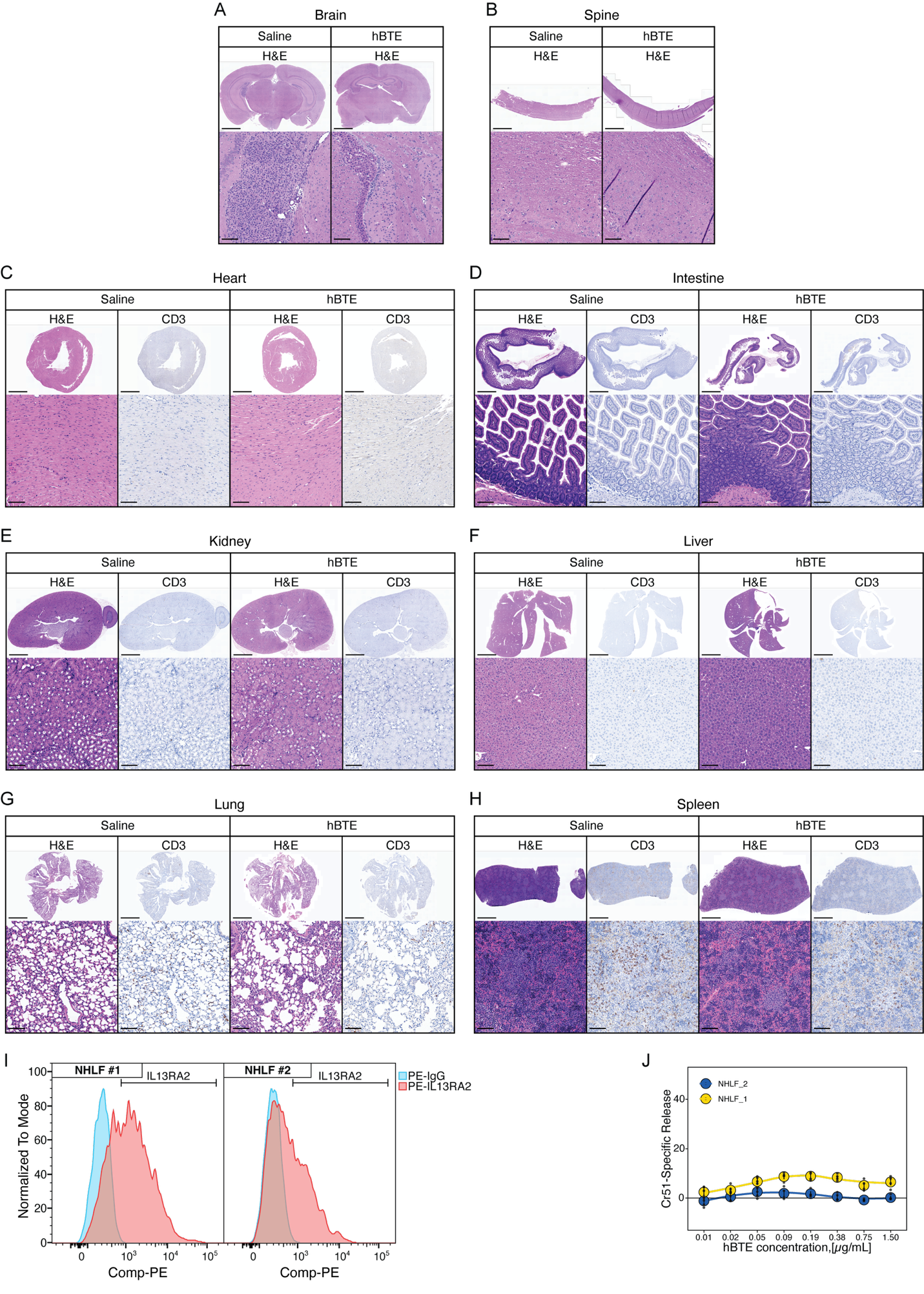
Supplemental Figure 3.** (A–H) Histopathological assessment of systemic and CNS toxicity following hBTE treatment in GBM6 PDX–bearing mice. (A–B) H&E staining of (A) brain and (B) spinal cord sections from saline (Left)- or hBTE (right)-treated mice, corresponding to the CD3, GFAP, and Luxol Fast Blue stains in Figure 6H (brain) and Figure 6I (spinal cord). Low magnification (top), scale bar = 1.5 mm; high magnification (bottom), scale bar = 100 µm. (C–H) H&E and CD3 immunohistochemical staining of (C) heart, (D) intestine, (E) kidney, (F) liver, (G) lung, and (H) spleen from saline- (left) or hBTE- (right) treated mice. Low magnification (top), scale bar = 1.5 mm; high magnification (bottom), scale bar = 100 µm. (I) Flow cytometric assessment of IL13RA2 surface expression on two primary normal human lung fibroblast (NHLF) samples derived from healthy donors, detected using a PE-conjugated anti-human IL13RA2 antibody. (J) Chromium-51 release assays demonstrating no T cell–mediated cytotoxicity against either NHLF sample following hBTE treatment (n = 4).

**
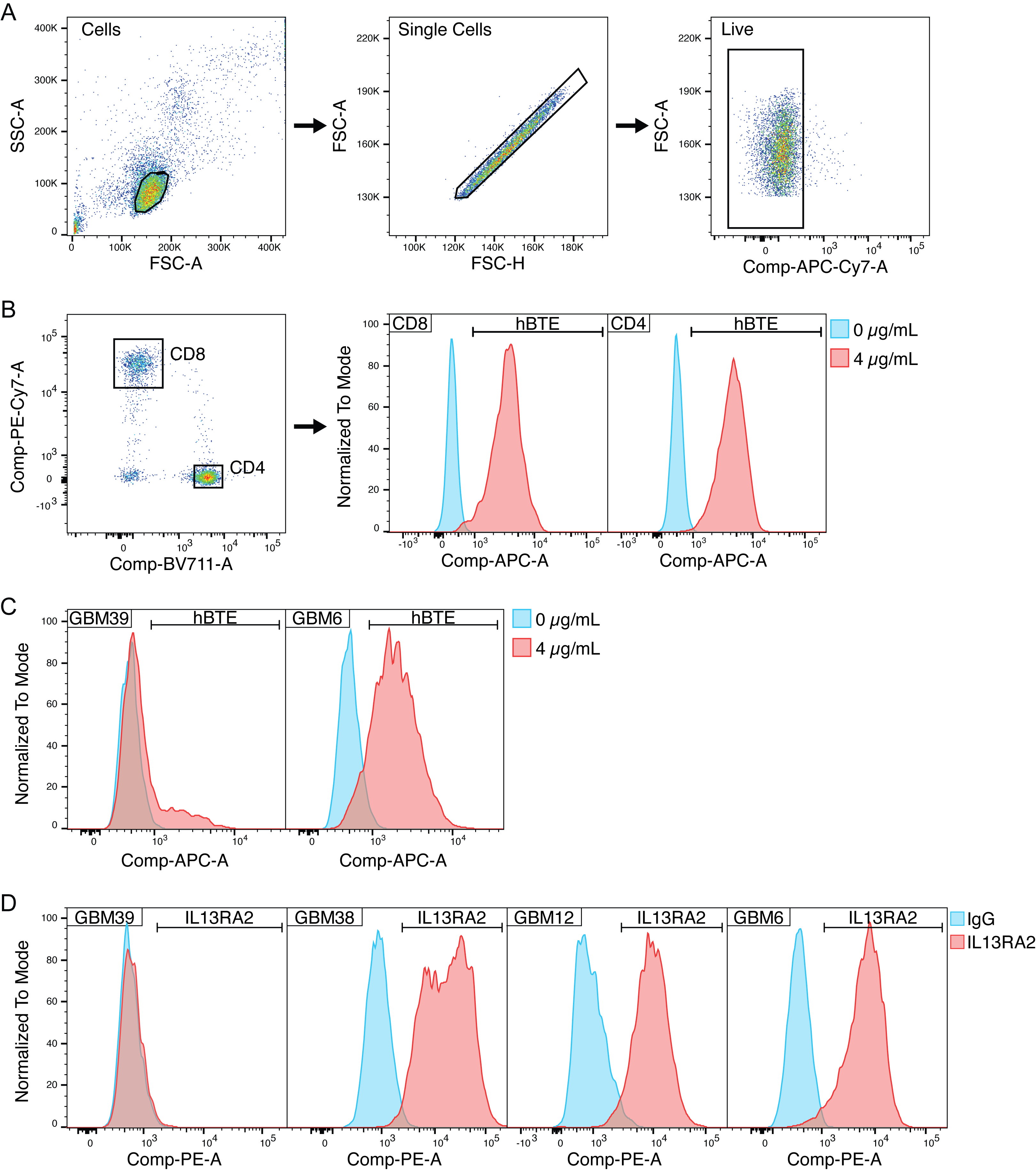
Supplemental Figure 4. Flow cytometry gating strategy.** (A) Standard gating strategy for all flow cytometry experiments, sequentially identifying cells, singlets, and live cells. (B) hBTE binding to CD8 and CD4 T cells, detected using an AF647-conjugated anti-6His tag antibody. (C) hBTE binding to GBM6 and GBM39 cells, detected using an AF647-conjugated anti-6His tag antibody. (D) IL13RA2 surface expression in patient-derived GBM cell lines GBM6, GBM12, GBM38, and GBM39, detected using a PE-conjugated anti-human IL13RA2 antibody or an PE-IgG control antibody.

**
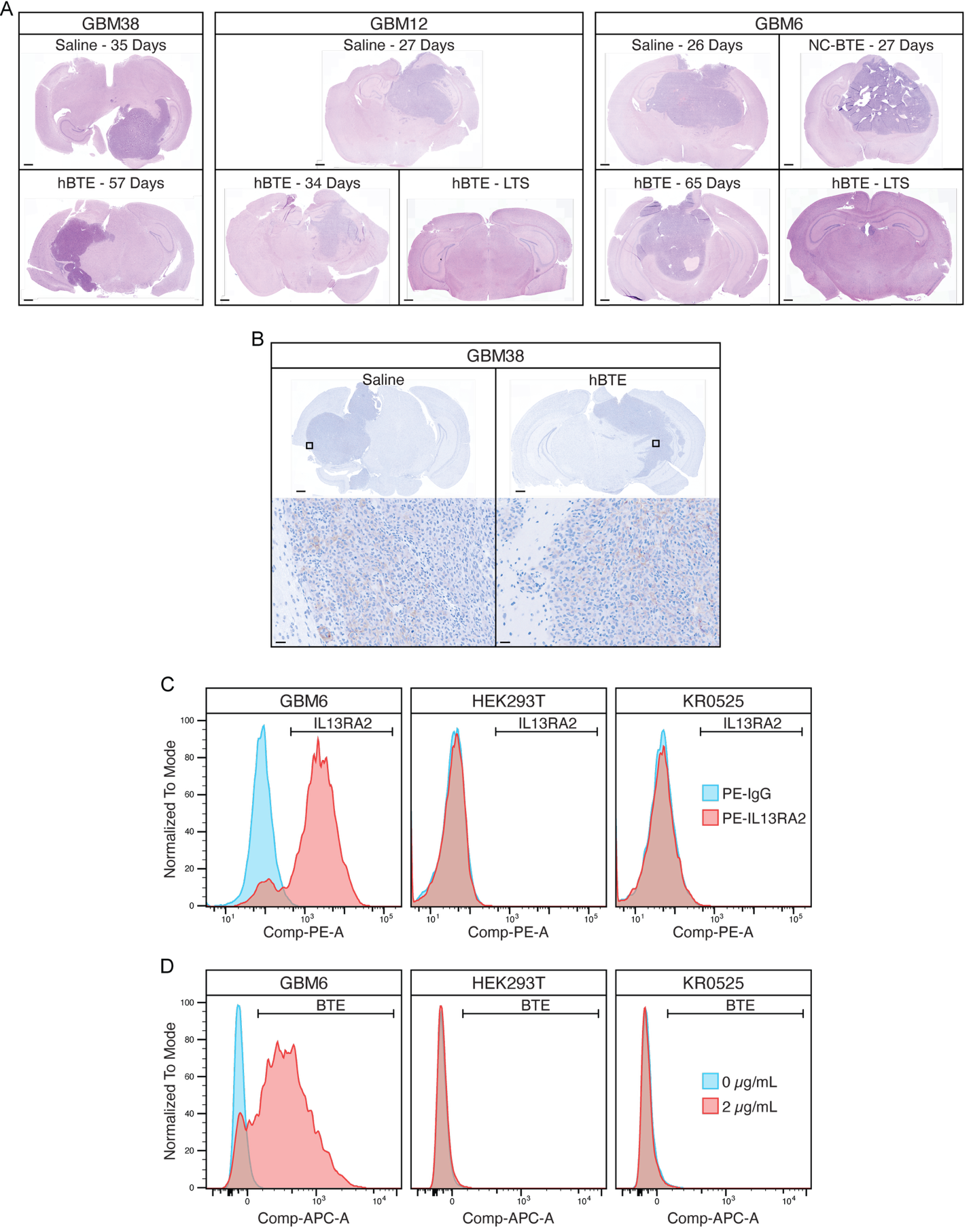
**

**Supplemental Figure 5. PD GBM tumor engraftment, IL13RA2 expression, and hBTE off-target binding to normal cells.** (A) Representative H&E staining of brain sections from PD-GBM–bearing mice at endpoint and from long-term surviving (LTS) mice following treatment with hBTE or control. Scale bars, 500 μm. (B) IHC staining for IL13RA2 of brain sections from GBM38–bearing mice at endpoint following hBTE or control treatment. Scale bars, 500 μm upper, 50 μm lower. (C) IL13RA2 surface expression in the patient-derived GBM cell line GBM6, immortalized renal cell line HEK293T, and normal human keratinocytes (NHKC), detected using a PE-conjugated anti-human IL13RA2 antibody or a PE-IgG control. (D) hBTE binding to GBM6, HEK293T, and NHKC cells, detected using an AF647-conjugated anti-6His tag antibody.
